# AcrSeek: metric learning with hybrid negative mining for anti-CRISPR protein detection under extreme class imbalance

**DOI:** 10.1093/bioinformatics/btag729

**Published:** 2026-09-29

**Authors:** Chan-Seok Jeong

**Affiliations:** Division of National Supercomputing, Korea Institute of Science and Technology Information, Daejeon, Republic of Korea

## Abstract

**Motivation:**

Anti-CRISPR (Acr) proteins inhibit CRISPR-Cas immunity and are key targets for precise control of CRISPR-based genome editing and phage–host coevolution research. Their experimental identification is costly and low-throughput, so computational predictors are used to prioritise candidates for testing. In this setting validated positives number in the hundreds while phage-derived putative negatives reach tens of thousands, producing extreme class imbalance. Yet existing classification-based predictors train and evaluate on 1:1 balanced datasets and allow high-similarity sequences between training and test, overestimating real-world performance.

**Results:**

We present AcrSeek, a metric-learning framework that trains a projection head over a frozen protein language model (PLM) encoder with hybrid negative mining (offline global plus online semi-hard) under a joint triplet–focal objective. Under nested 5-fold cross-validation with leakage controlled by 40% identity clustering, AcrSeek improved AUPRC on the imbalanced (1:383) test set from 0.017 to 0.355 over AcrNET (matched PLM backbone), reaching 0.508 with ESM-2 3B. AcrSeek therefore remains structurally robust under extreme class imbalance and may also serve other protein-function detection problems with scarce positives.

**Availability and implementation:**

Source code at https://github.com/jeongchans/acrseek, archived at Zenodo (DOI: 10.5281/zenodo.21946803); data and model checkpoints at Zenodo (DOI: 10.5281/zenodo.20115442).

## 1 Introduction

CRISPR-Cas systems are the adaptive immune machinery of prokaryotes, recognising and cleaving foreign nucleic acids in a sequence-specific manner (Makarova *et al*. 2020). To counter this immunity, phages have evolved anti-CRISPR (Acr) proteins that inhibit CRISPR-Cas activity (Bondy-Denomy *et al*. 2013), and the resulting defence–anti-defence co-evolution is a central driver of the prokaryote–phage arms race (Pawluk *et al*. 2018). Acr proteins are also increasingly attractive as tools for the precise control of CRISPR-based genome editing (Marino *et al*. 2020), making the systematic discovery of new Acrs important for both basic biology and downstream applications.

Because experimental identification of Acrs is costly and low-throughput, computational predictors have been actively developed. PaCRISPR (Wang *et al*. 2020) combined sequence-derived features with classical machine learning, DeepAcr (Wandera *et al*. 2022) scored each protein from a one-hot encoding of its sequence together with whole-protein physicochemical properties, and AcrNET (Li *et al*. 2023) subsequently achieved improved performance using a deep neural network on pretrained protein language model (PLM) embeddings. All three, however, were trained and evaluated on datasets balanced 1:1 between positives and negatives, and the positive-side clustering thresholds applied by PaCRISPR and AcrNET (70% identity and 95%, respectively) still leave open the possibility of sequence leakage between training and test sets. AcRanker (Eitzinger *et al*. 2020) sidesteps the balancing constraint by performing pairwise ranking within each proteome, but relies on 412-dimensional handcrafted *k*-mer features whose representational capacity is limited compared with PLM embeddings. Gussow *et al*. (2020) departs from all of these by scoring genomic context rather than the protein sequence itself, using a random forest to nominate 2500 candidate Acr families.

Beyond these methodological choices, the intrinsic difficulty of Acr detection stems from a profound asymmetry in data scale. anti-CRISPRdb (Dong *et al*. 2018) currently lists about 1400 experimentally supported Acr entries, which collapse to only a few hundred representative sequences after sequence-identity-based redundancy reduction. Phage-derived negative candidates, by contrast, number in the tens of thousands even after the same procedure, yielding class ratios on the order of several hundredfold. Evaluating classifiers trained on 1:1 balanced data therefore systematically inflates precision relative to deployment, and to our knowledge no prior work has assessed Acr predictors under realistic class priors.

To address this extreme imbalance, we propose AcrSeek, a metric learning framework for Acr detection. The key idea is to bypass the limitations of classification-based approaches by recasting detection as distance learning, and to integrate a negative mining strategy and loss combination tailored to severe imbalance. Metric learning has been adopted in adjacent biological problems, including enzyme function prediction (Yu *et al*. 2023) and drug–target interaction prediction (Yang *et al*. 2024); here we specialise it for Acr detection under extreme class imbalance. AcrSeek places a trainable projection head on top of a frozen PLM encoder (Rives *et al*. 2021, Lin *et al*. 2023) and trains the head with hybrid negative mining—offline global candidate selection combined with online semi-hard mining—under a joint objective that combines triplet (Schroff *et al*. 2015) and focal (Lin *et al*. 2017) losses.

For evaluation, we apply a nested 5-fold cross-validation protocol that controls leakage through 40% identity clustering and assesses performance at a realistic class ratio of approximately 1:383. On the same dataset and splits, we systematically compare AcrSeek against AcrNET and AcRanker and quantify class-ratio robustness through BLAST E-value-based difficulty stratification. We further examine performance across PLM scales (ESM-1b 650M, ESM-2 650M, ESM-2 3B) and probe residue-level interpretability through an AcrIF11 case study. Concretely, AcrSeek improves AUPRC over AcrNET on the imbalanced test set by approximately 21-fold under controlled comparison and by about 30-fold when scaled to ESM-2 3B, demonstrating the robustness of the metric learning approach under extreme class imbalance.

## 2 Materials and methods

Figure 1 provides an overview of the AcrSeek pipeline.

**Figure 1:**
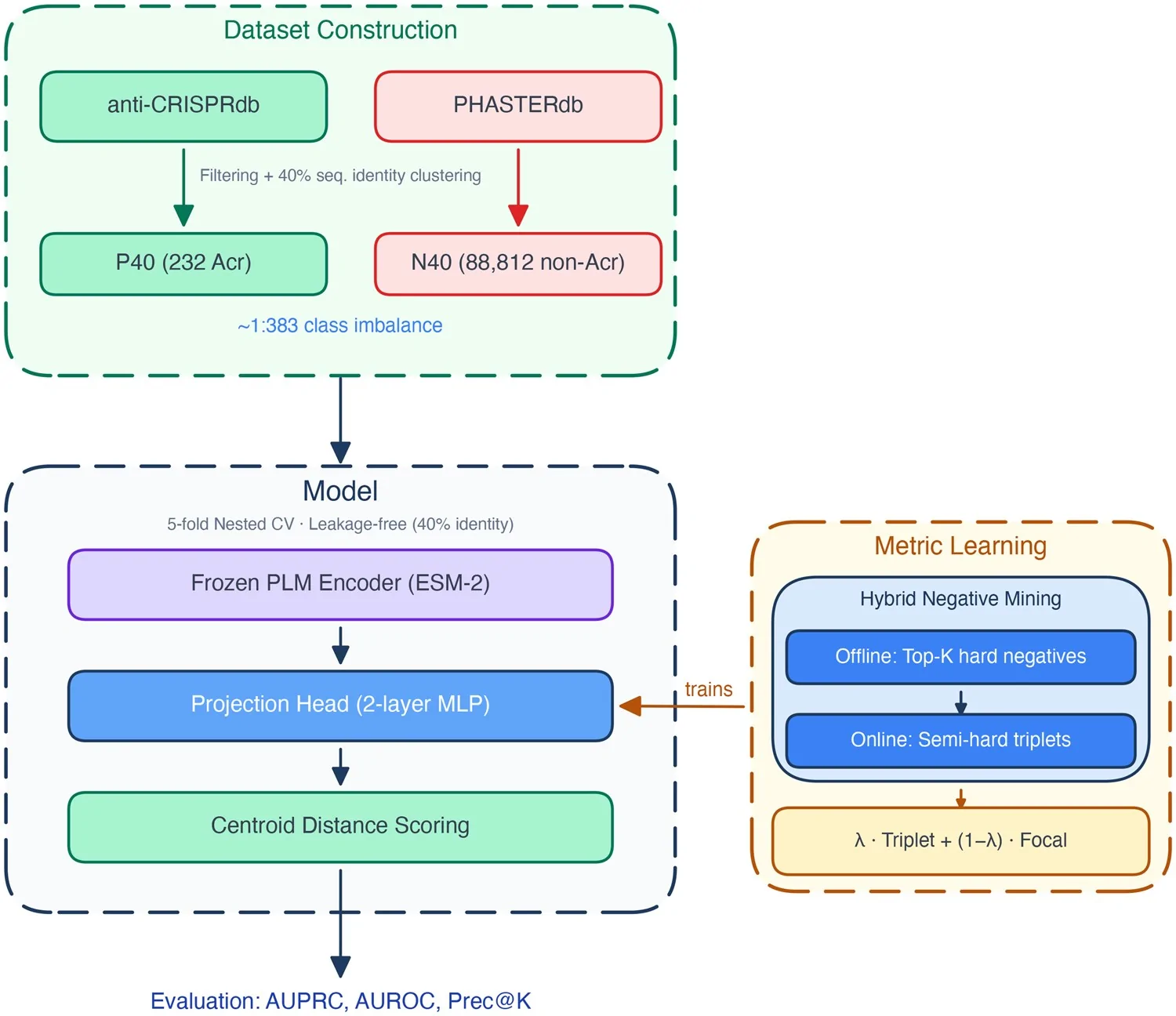
Overview of the AcrSeek framework. The extreme-imbalance dataset is built from anti-CRISPRdb and PHASTERdb through 40% sequence-identity clustering. Each input sequence is projected into the metric space by a frozen PLM encoder and a trainable projection head, and Acr membership is decided from its distance to the positive centroid. The projection head is trained with metric learning under offline–online hybrid negative mining.

### 2.1 Datasets

We learn a binary detection function f(x)∈[0,1] that classifies a protein sequence *x* as Acr (y=1) or non-Acr (y=0). The positive set P consists of experimentally validated Acrs and the negative set N of prophage-derived proteins. The latter are putative negatives: no experimentally verified non-Acr set exists at this scale, so N is assembled by excluding sequences that resemble known Acrs rather than by establishing that its members lack Acr activity. We use “negatives” as shorthand for these putative negatives throughout, and §4 sets out the consequences. Figure 2 summarises the dataset construction pipeline.

**Figure 2:**
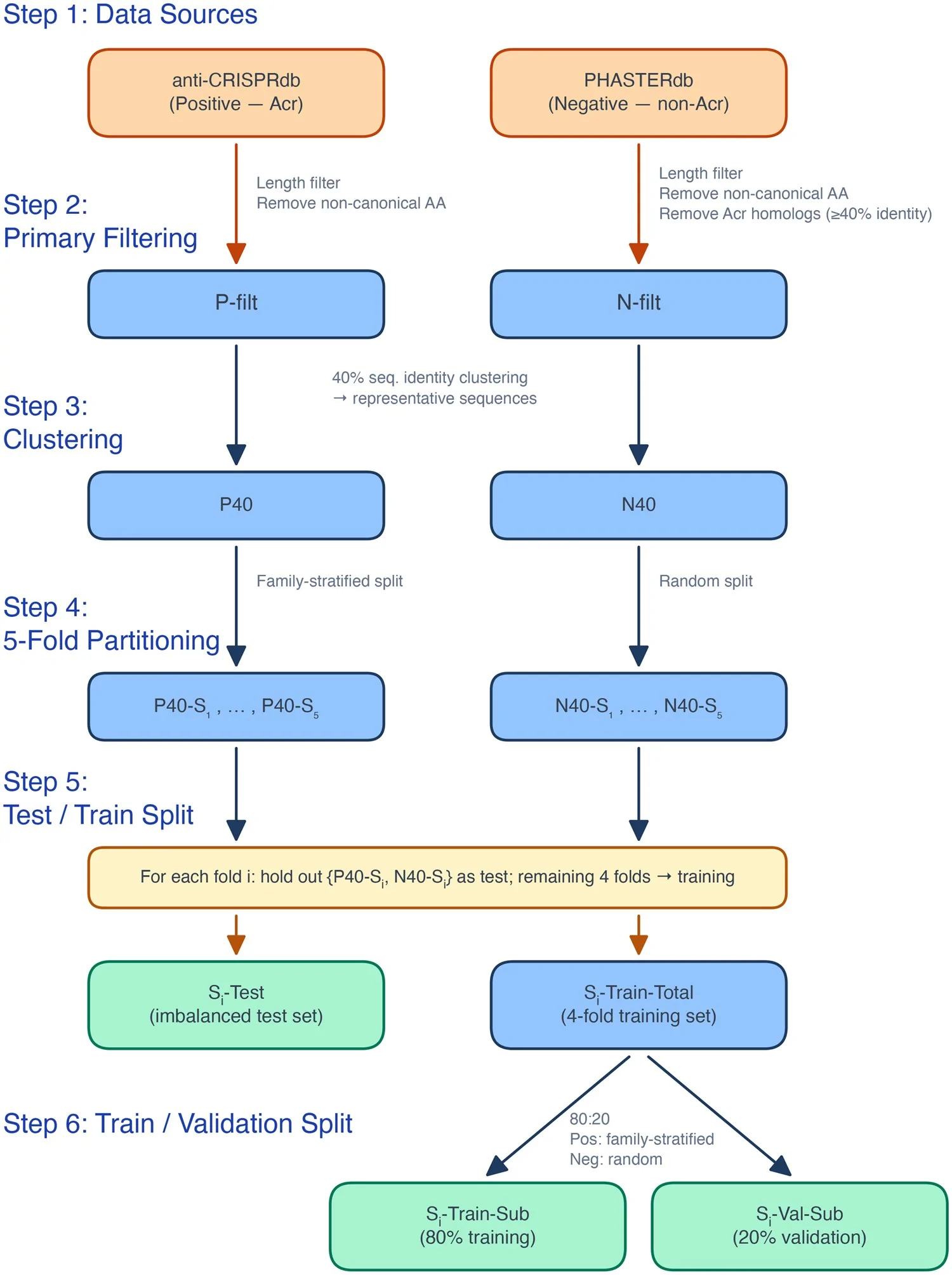
Dataset construction pipeline. Source databases are filtered, clustered, and split into folds to produce the training, validation, and test sets.

#### 2.1.1 Positive set (P40)

The positive data start from 1378 experimentally supported entries in anti-CRISPRdb (Dong *et al*. 2018) and pass through two filtering stages. The first stage applies (i) sequence length 30–350 aa, (ii) removal of non-standard amino acids, and (iii) ID and sequence deduplication, yielding P-filt with 1154 sequences (the length range follows PaCRISPR (Wang *et al*. 2020) and AcrNET (Li *et al*. 2023)). The second stage clusters P-filt with MMseqs2 (Steinegger and Söding 2017) at 40% sequence identity (coverage ≥ 0.8), retains the longest sequence in each cluster as the representative, and produces the final P40 set (232 sequences from 86 Acr families). P40 covers 54–322 aa, with a long-tailed family distribution in which the top five families account for roughly 54% of all sequences.

#### 2.1.2 Putative negative set (N40)

The negative data start from 418 113 prophage-derived proteins in PHASTERdb (Arndt *et al*. 2016). We apply the same length and amino-acid filters and ID/sequence deduplication as for the positives, and use MMseqs2 to remove 157 potential Acr homologues sharing ≥ 40% identity with any anti-CRISPRdb positive, yielding N-filt with 250 227 sequences. Clustering at 40% identity then produces the final N40 set (88 812 sequences). The resulting P40: N40 ratio is 232:88 812, giving an extreme class imbalance of approximately 1:383.

#### 2.1.3 Data splitting

P40 and N40 are combined into a 5-fold cross-validation dataset. Positives are distributed across folds by family-stratified greedy bin-packing (families sorted by size in descending order and assigned to the currently smallest fold) to keep family proportions uniform, while negatives are split uniformly at random. For each fold $S_{i}$, the test set ($S_{i}$-Test) is fold *i* and the four remaining folds form the total training set ($S_{i}$-Train-Total), which is further split 80:20 into a training subset ($S_{i}$-Train-Sub) and a validation subset ($S_{i}$-Val-Sub). Table 1 reports the split statistics.

**Table 1:** Dataset split statistics.

Split	P40	N40	Total
$S_{i}$-Test	46–47	17 762–17 763	17 808–17 810
$S_{i}$-Train-Sub	148–149	56 839–56 840	56 987–56 989
$S_{i}$-Val-Sub	37	14 210	14 247
All (P40 + N40)	232	88 812	89 044

### 2.2 Model architecture

AcrSeek is a two-stage model that stacks a frozen protein language model (PLM) encoder with a trainable projection head (Fig. 1).

#### 2.2.1 Frozen PLM encoder

For an input sequence $x=(x_{1},\ldots ,x_{L})$, the PLM produces residue-level representations $\{h_{i}\}\in R^{L\times D}$, which we mean-pool into a sequence embedding $e=\frac{1}{L}\sum_{i=1}^{L}h_{i}\in R^{D}$. The PLM parameters are kept frozen during training. ESM-2 650M (Lin *et al*. 2023) is used as the primary encoder, and we additionally compare against ESM-1b 650M (Rives *et al*. 2021) and ESM-2 3B.

#### 2.2.2 Projection head

The PLM embedding $e\in R^{D}$ is mapped into a lower-dimensional metric space by a two-layer MLP:


(1) $$z=\frac{g(e)}{\|g(e){\|}_{2}},\;g(e)=W_{2}\text{ReLU}(W_{1}e+b_{1})+b_{2}$$

where $W_{1}\in R^{d\times D}$ and $W_{2}\in R^{d\times d}$. The output z is L2-normalised onto the unit hypersphere, which prevents the trivial solution of inflating vector magnitudes and constrains pairwise distances to [0,2], simplifying the interpretation of the margin *m*.

### 2.3 Training objective

To combine metric and classification signals, we train the projection head with a joint triplet–focal objective.

#### 2.3.1 Triplet loss

For an anchor $z_{a}$, a positive $z_{p}$, and a negative $z_{n}$, we apply the triplet margin loss


(2) $$L_{\text{triplet}}=\frac{1}{\left|T\right|}\sum_{(a,p,n)\in T}\text{max}\left(0,\;\|z_{a}-z_{p}{\|}_{2}-\|z_{a}-z_{n}{\|}_{2}+m\right),$$

where m>0 is the margin and T is the set of semi-hard triplets selected by online mining (§2.4). Because Euclidean and cosine distance are monotonically related on L2-normalised vectors, we use the standard Euclidean distance throughout.

#### 2.3.2 Focal loss

We use focal loss (Lin *et al*. 2017) as an auxiliary classification signal that supervises every sample in the batch. Originally proposed to address extreme class imbalance in object detection, focal loss has since been shown to be effective in other highly imbalanced settings such as medical imaging (Abraham and Khan 2019). Classification probabilities are obtained by passing the distance to the positive centroid μ, $d=\|z-\mu {\|}_{2}$, through a learnable sigmoid:


(3) p=σ(κ·(c−d)),

where κ>0 is a temperature and *c* is a decision boundary, both learnable scalars. This distance-based probability shares the same metric as the triplet loss, and the same centroid distance is reused at inference, ensuring train–inference consistency. The focal loss is then


(4) $$L_{\text{focal}}=-\frac{1}{B}\sum_{i=1}^{B}\alpha_{t}{\left(1-p_{t}\right)}^{\gamma }\;\text{log}\;p_{t},$$

with $p_{t}=p\cdot y+(1-p)(1-y)$ and $\alpha_{t}=\alpha \cdot y+(1-\alpha )(1-y)$; γ is the focusing parameter and α the class weight.

#### 2.3.3 Combined loss


(5) $$L=\lambda L_{\text{triplet}}+(1-\lambda )L_{\text{focal}}$$

The mixing weight λ∈[0,1] balances the two losses: $L_{\text{triplet}}$ shapes the pairwise distance structure of the embedding space, while $L_{\text{focal}}$ acts on the centroid-distance-based classification probability. When no valid semi-hard triplet exists in a batch, $L_{\text{triplet}}$ is skipped and training proceeds with $L_{\text{focal}}$ alone.

### 2.4 Hybrid negative mining

Rather than using all negatives in every batch under the extreme imbalance, we combine two mining stages: a global (offline) stage that selects hard-negative candidates near the positive centroid (Wu *et al*. 2017), and a local (online) stage that performs semi-hard triplet mining within each batch (Schroff *et al*. 2015).

#### 2.4.1 Offline mining (global)

At the start of each epoch we re-project all negatives with the current projection head and select the *K* negatives with the smallest Euclidean distance to the positive centroid μ as the hard-negative candidate pool $N_{K}\subset N$. The centroid is defined as the L2-normalised mean of the training-positive projections at that moment:


(6) $$\mu =\frac{1}{\left|P_{\text{train}}\right|}\sum_{x\in P_{\text{train}}}\frac{z_{x}}{\|z_{x}{\|}_{2}}.$$

We set $K=n_{\text{batches}}\times n_{\text{neg}/\text{batch}}\times r$ (with r=10 fixed), which reduces the effective negative set from |N| to $\left|N_{K}\right|$.

#### 2.4.2 Online mining (local)

We then form 1:1 balanced batches by pairing positives with negatives drawn from $N_{K}$. For every anchor–positive pair (i,j) in a batch, we pick the negative satisfying the semi-hard condition


(7) $$\|z_{a}-z_{p}{\|}_{2}<\|z_{a}-z_{n}{\|}_{2}<\|z_{a}-z_{p}{\|}_{2}+m$$

that is closest to the anchor (the hardest semi-hard negative). Hard triplets (d(a,n)<d(a,p)) are deliberately excluded: the small positive set leaves training vulnerable to outliers in early epochs, and because offline-selected candidates already lie near the centroid, in-batch hard negatives are likely to be noise (Schroff *et al*. 2015). As training progresses, formerly hard triplets gradually enter the semi-hard region. Algorithm 1 summarises the full training loop.

Algorithm 1 Hybrid negative mining training loop

**Req** **uire:** Positive set P, negative set N, frozen PLM embeddings {(id,e)}, projection head $g_{\theta }$, distance classifier (κ,c)

1: **for** epoch =1,2,…,E  **do** 

2:  Project: $z_{x}\leftarrow g_{\theta }(e_{x})/\|g_{\theta }(e_{x})\|$  ∀x∈P∪N

3:  Update centroid: $\mu \leftarrow \text{mean}({\left\{z_{i}\right\}}_{i\in P})$, L2-normalized

4:  **Offline mining:**  $N_{K}\leftarrow \text{top}-K(N,\;\mu )$

5:  **for** each balanced batch $B=(B_{p},B_{n})\subset P\times N_{K}$  **do** 

6:   $Z_{p}={\left\{z_{i}\right\}}_{i\in B_{p}}$,  $Z_{n}={\left\{z_{j}\right\}}_{j\in B_{n}}$

7:   **Online mining:**  $T\leftarrow \text{SemiHardTriplets}(Z_{p},Z_{n},m)$

8:   $L_{\text{tri}}\leftarrow \text{TripletLoss}(T,m)$ if T≠∅ else 0

9:   Distance: $d_{i}\leftarrow \|z_{i}-\mu {\|}_{2}$  ∀i∈B

10:   Probability: $p_{i}\leftarrow \sigma (\kappa \cdot (c-d_{i}))$

11:   $L_{\text{foc}}\leftarrow \text{FocalLoss}(\{p_{i}\},\{y_{i}\},\gamma ,\alpha )$

12:   $L\leftarrow \lambda L_{\text{tri}}+(1-\lambda )L_{\text{foc}}$

13:   θ,κ,c←AdamW(∇L)

15:  **end for** 

16: **end for** 

### 2.5 Inference

After training, inference reuses the same distance metric as training. A new sequence *x* is embedded by the PLM, projected by the head, L2-normalised, and converted to a probability through the centroid-distance sigmoid:


(8) $$\hat{p}(x)=\sigma \left(\kappa \cdot (c-\|z_{x}-\mu {\|}_{2})\right).$$

This score is produced by the same pathway as the focal-loss classification probability during training.

### 2.6 Evaluation protocol

#### 2.6.1 Nested 5-fold cross-validation

We use nested 5-fold cross-validation to estimate generalisation performance while removing hyperparameter (HP) selection bias. For each outer fold $S_{i}$, the inner loop selects HPs and the outer loop evaluates the resulting model on the held-out test set.

The inner loop trains on $S_{i}$-Train-Sub and validates on $S_{i}$-Val-Sub, performing a grid search over four hyperparameters—the learning rate η, projection dimension *d*, loss-mixing weight λ, and triplet margin *m*—with the remaining settings fixed to standard values from the literature (§2.8). Training runs for a fixed number of epochs *E*, with validation AUPRC recorded at every 10 epochs; the optimal epoch is the one that maximises validation AUPRC after smoothing with a centred moving average (window 5), which mitigates the staircase shape of PR curves caused by the small number of validation positives. The outer loop then trains on $S_{i}$-Train-Sub with the selected HPs and epoch and evaluates on $S_{i}$-Test. Final performance is reported as the 5-fold mean ± standard deviation. Our protocol is a modification of standard nested cross-validation in two respects: the outer loop trains on $S_{i}$-Train-Sub rather than $S_{i}$-Train-Total, and HPs are shared across outer folds rather than selected per fold (Shared HP strategy; §2.8).

#### 2.6.2 Metrics

The primary metric is AUPRC (area under the precision–recall curve). Under extreme imbalance, AUROC is known to lose discriminative power as the negative class grows (Davis and Goadrich 2006, Saito and Rehmsmeier 2015), motivating AUPRC as the primary metric. We additionally report AUROC, Precision@*K* (with *K* equal to the number of test positives, which makes it equivalent to Recall@*K*), and FPR at TPR = 0.95. For threshold-based assessment we report Fixed F1 and Oracle F1: Fixed F1 measures operational performance at a single deployment threshold—the median of the per-fold inner-loop F1-maximising thresholds—applied uniformly to all folds, while Oracle F1 reports the upper bound obtained by maximising F1 on the test set itself.

### 2.7 Baselines

#### 2.7.1 AcrNET

AcrNET (Li *et al*. 2023) fuses three input streams—PLM embedding, evolutionary, and CNN-encoded local-structure features—through a fully-connected classification head, and is trained with cross-entropy. To control for input representation, we use the ESM-1b-only variant (Linear(1280, 256)–ReLU–Linear(256, 128)–ReLU–Linear(128, 2)) as our comparison target. Because the original code could not run directly due to environment and data compatibility issues, we reimplemented the architecture, training loop, and HPs to match the original specification, and confirmed that the reproduction reaches performance equivalent to the reported values on the balanced validation set (Supplementary Section S3, available as supplementary data at *Bioinformatics* online).

Since AcrNET is trained on a 1:1 balanced dataset, our imbalanced training data cannot be used directly, so we construct two balanced training sets. (1) $S_{i}$-Train-Bal reproduces the original protocol: positives are taken from P-filt after removing sequences with ≥ 90% identity to $S_{i}$-Test (allowing up to 90% train–test similarity), and negatives are paired 1:1 from the PaCRISPR source (Wang *et al*. 2020). (2) $S_{i}$-Train-Sub-Bal serves as a controlled comparison: it draws from the same P40 + N40 source as AcrSeek by random subsampling of negatives at 1:1, with train–test positive between-similarity (the maximum pairwise sequence identity between any training positive and any test positive) capped at 40%. AcrNET(90%) and AcrNET(40%) are the models trained on (1) and (2), respectively. The published AcrNET weights could not serve either track: the released checkpoint is for the full three-stream model rather than the ESM-1b-only variant compared here, and a single checkpoint trained on anti-CRISPRdb—from which our positives are also drawn—supplies none of the per-fold leakage control that both tracks impose. We use the paper’s HPs (steps = 3000, lr = 0.001, batch = 16, Adam) as the default and also pre-validated the original grid (steps ∈{30,300,3,000}, lr ∈{0.1,0.01,0.001,0.0001}) on both training tracks. The paper’s HPs were optimal for AcrNET(90%), which reproduces the original protocol, while lr = 0.01 was selected for AcrNET(40%), which controls for positive leakage (Supplementary Section S3, available as supplementary data at *Bioinformatics* online).

#### 2.7.2 AcRanker

AcRanker (Eitzinger *et al*. 2020) is an XGBoost-based pairwise ranking model that takes a 412-dimensional feature vector composed of amino-acid composition and dimer/trimer frequencies based on physicochemical groups. We apply the pretrained model from the public repository directly—without retraining—to score the test sequences and evaluate them with the same metrics as the other methods. Fixed F1 is not reported for AcRanker because it requires calibrated probabilities.

### 2.8 Implementation details

Table 2 summarises the training configuration. The four searched hyperparameters—learning rate η, projection dimension *d*, loss weight λ, and margin *m*—together with the number of epochs *E* are selected per encoder in the inner loop on the basis of validation AUPRC (search ranges are listed in Supplementary Section S1), while the remaining items are fixed across all embedding variants to standard values from the literature or to predetermined defaults. Because each inner-loop validation set contains only about 37 positives per fold, fold-independent HP selection—choosing the per-fold maximum across the 54 candidate combinations—is vulnerable to the winner’s curse driven by validation noise. To mitigate this risk, we adopt a Shared HP strategy: for each embedding we compute the 5-fold mean validation AUPRC of every one of the 54 (HP, epoch) combinations, select a single combination on this aggregate basis, and apply it uniformly to all outer folds. Because the aggregate is formed over all five inner folds, HP selection is coupled across outer folds; Supplementary Section S1 describes this coupling and compares the two selection rules empirically. All experiments were performed on a single NVIDIA RTX PRO 6000 (Blackwell, 96 GB) GPU. For efficiency, PLM embeddings were pre-extracted so that only the projection head is trained; this requires approximately four minutes per fold even for the largest ESM-2 3B encoder.

**Table 2:** Training configuration.

Setting	Symbol	ESM-1b	ESM-2	ESM-2
		650M	650M	3B
*Searched (selected via inner loop)*				
Embedding dim.	*D*	1280	1280	2560
Projection dim.	*d*	128	256	512
Learning rate	η	$2\times 10^{-5}$	$3\times 10^{-5}$	$5\times 10^{-5}$
Loss weight	λ	0.3	0.5	0.7
Margin	*m*	0.5	1.0	0.5
Epochs	*E*	800	260	320
*Fixed (shared across encoders)*				
Optimizer			AdamW	
Weight decay			$1\times 10^{-4}$	
Batch composition			pos 32 + neg 32	
Offline multiplier	*r*		10	
Focal γ			2.0	
Focal α			0.25	
κ init			5.0	
*c* init			1.0	
Random seed			42	

The top block lists hyperparameters selected by the per-encoder search; the bottom block lists settings fixed across all encoders.

### 2.9 BLAST-based difficulty stratification

To quantify test-sample difficulty, we use blastp from BLAST+ (Altschul *et al*. 1990) as a sequence-homology measure. For each fold, the entire training-positive set ($S_{i}$-Train-Total) is built into a database, and the test sequences are queried against it; the best-hit *E*-value serves as the difficulty score. We use default parameters (*E*-value cutoff 10, BLOSUM62) and keep a separate database per fold to preserve the cross-validation structure. On the basis of *E*-value, test samples are stratified into a strong group (*E*-value $<10^{-3}$; significant homology to known Acrs) and a weak group (*E*-value $\geq 10^{-3}$; weak or insignificant homology), with the same criterion applied to both positives and negatives.

## 3 Results

We assess AcrSeek along four axes: (i) comparison against existing baselines (§3.1); (ii) robustness to changes in class ratio and sequence homology (§3.2, §3.4); (iii) mechanistic analysis of loss composition and embedding dynamics (§3.3, §3.6); and (iv) residue-level interpretability (§3.7).

### 3.1 Performance on the imbalanced test set

Table 3 reports 5-fold mean performance on the imbalanced test set (approximately 1:383), and Fig. 3 shows the corresponding precision–recall curves; per-fold metrics are in Supplementary Section S2, available as supplementary data at *Bioinformatics* online.

**Figure 3:**
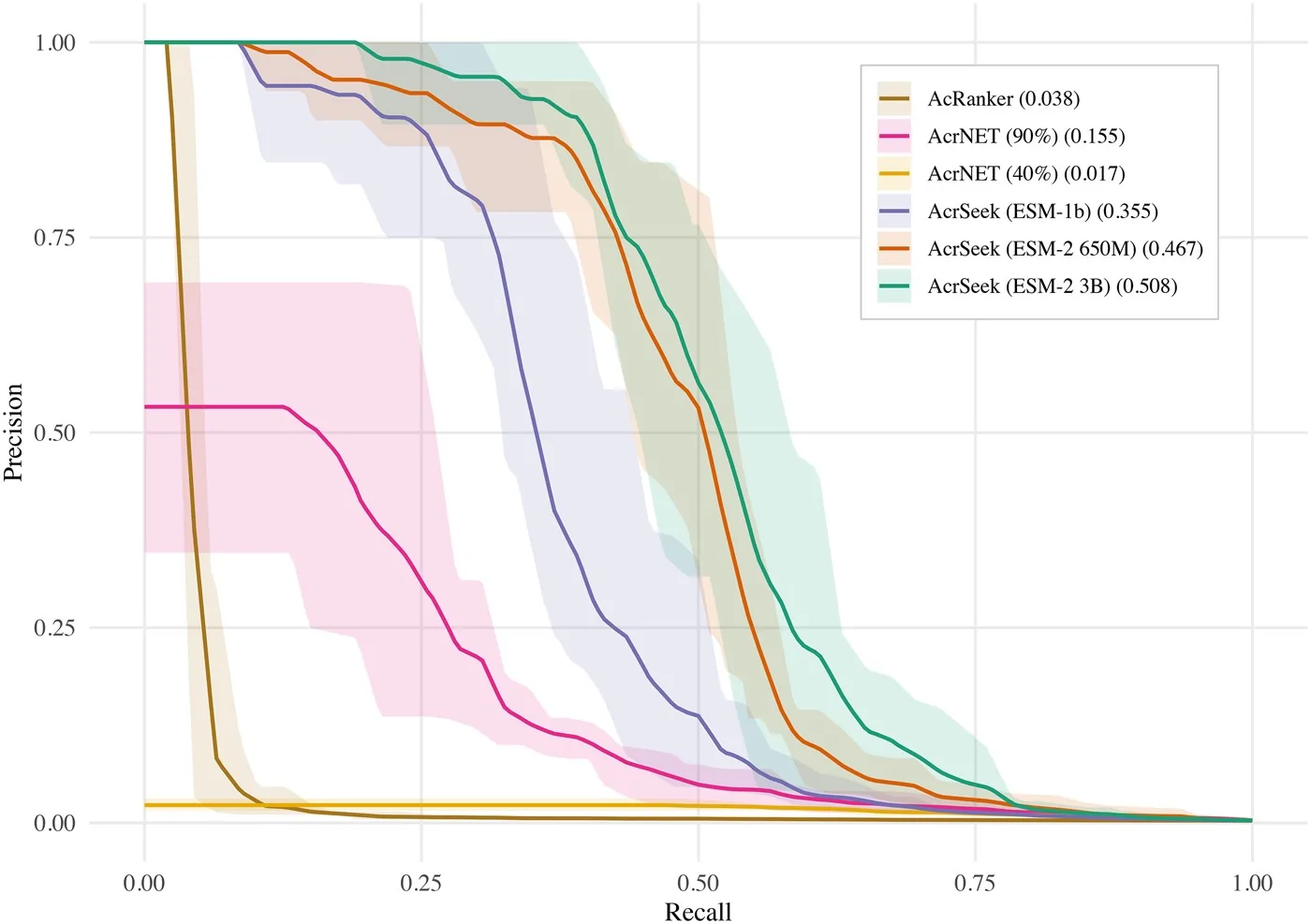
Precision–recall curves on the imbalanced test set. Solid lines show the 5-fold mean and the shaded band the across-fold range; AUPRC values appear in parentheses in the legend. Each curve begins at the first operating point the model can actually reach: where many top-ranked sequences share one score, no threshold separates them, and the precision attainable over that range is the flat value shown. This is why AcrNET(40%), whose positive-class probability saturates at 1.0 for on average 1010 sequences per fold, is flat at ∼0.02 up to recall ∼0.45.

**Table 3:** Performance comparison on the imbalanced test set.

Method	AUPRC	AUROC	Prec@K	FPR@95	Fixed F1	Oracle F1
AcRanker	0.0381 (0.0090)	0.6455 (0.0455)	0.0475 (0.0239)	0.8726 (0.0453)	—^a^	0.0759 (0.0235)
AcrNET (90%)	0.1549 (0.0475)	0.8952 (0.0174)	0.2547 (0.0490)	**0.4213** (0.1129)	0.0288^b^ (0.0156)	0.2815 (0.0534)
AcrNET (40%)	0.0171 (0.0035)	0.8625 (0.0244)	0.1464 (0.0632)	0.5781^c^ (0.1161)	0.0179^b^ (0.0009)	0.0434 (0.0101)
AcrSeek (ESM-1b 650M)	0.3547 (0.0524)	0.8662 (0.0234)	0.3665 (0.0389)	0.6447 (0.1264)	0.4393 (0.0341)	0.4563 (0.0325)
AcrSeek (ESM-2 650M)	0.4673 (0.0355)	**0.9055** (0.0203)	0.4914 (0.0314)	0.4810 (0.1182)	0.5236 (0.0278)	0.5554 (0.0440)
AcrSeek (ESM-2 3B)	**0.5077** (0.0523)	0.8999 (0.0285)	**0.5045** (0.0448)	0.5585 (0.1720)	**0.5248** (0.0334)	**0.5789** (0.0308)

5-fold mean (standard deviation). Bold indicates the best value in each column. For FPR@95 the best value is the lowest.

a AcRanker does not produce a fixed threshold, so Fixed F1 cannot be computed.

b AcrNET uses the default threshold 0.5 without explicit calibration; AcrSeek’s Fixed F1 uses a single deployment threshold computed as the median of per-fold inner-loop thresholds, so direct comparison should be made with care. ^c^For AcrNET(40%), only about 70% of positives are well separated at prob ≈ 1, while the remaining positives fall into a prob-≈-0 noise-floor region tied with many negatives. Because reaching TPR = 0.95 (45/47) requires including some of these noise-floor positives, the operative threshold lies in a flat, tied region, making the resulting FPR value unstable.

AcRanker is the weakest baseline, with an AUPRC of 0.038; its AUROC of 0.646 is above the random level (0.5) but its precision–recall behaviour is not at a practical detection level.

AcrNET(90%), trained under the original protocol that allows up to 90% identity between training and test sets, achieves AUPRC 0.155 and Precision@*K* 0.255. AcrSeek (ESM-2 3B) reaches AUPRC 0.508, which is 3.3× higher than AcrNET(90%), and AcrSeek (ESM-1b, 0.355), which shares ESM-1b embeddings with AcrNET, still shows a 2.3× gap. Since AcrNET(90%) benefits from a more permissive leakage condition, this comparison gives a conservative estimate of AcrSeek’s advantage. AcrNET(40%), retrained with leakage controlled at 40% identity, drops to AUPRC 0.017—roughly nine times lower than AcrNET(90%). Under matched 40% leakage control and ESM-1b embeddings, AcrSeek (ESM-1b, 0.355) exceeds AcrNET(40%, 0.017) by approximately 21×, and with the larger encoder AcrSeek (ESM-2 3B, 0.508) widens the gap to about 30×. With both the PLM and the leakage condition controlled, this gap shows that the performance difference comes from the loss function and training strategy rather than from the input representation.

For FPR at TPR = 0.95, AcrNET(90%, 0.421) is better than the AcrSeek variants (0.481–0.645). The reported value for AcrNET(40%) is 0.578, but it is unstable because a fraction of positives is grouped together with the negatives in a probability-≈-0 noise-floor region (Table 3, footnote c). By contrast, Precision@*K*, which directly measures the precision of the top candidates, shows that AcrSeek (ESM-1b, 0.367) outperforms AcrNET(40%, 0.146) by 2.5×.

Within AcrSeek, performance increases consistently with the PLM encoder. AUPRC follows ESM-1b 650M (0.355) → ESM-2 650M (0.467) → ESM-2 3B (0.508), and the same trend holds for Precision@K (0.367 → 0.491 → 0.505) and Oracle F1 (0.456 → 0.555 → 0.579). The gain from ESM-1b 650M to ESM-2 650M (+0.112) reflects improved pretraining at the same model scale (650M parameters), while ESM-2 650M to ESM-2 3B (+ 0.041) reflects scaling under a fixed pretraining recipe; the metric learning framework therefore propagates both kinds of PLM quality improvement.

The results above use the Shared HP strategy (§2.8). To assess how far they depend on that choice, we repeated the outer loop for all three encoders with fold-independent HPs (Supplementary Section S1, available as supplementary data at *Bioinformatics* online). Test AUPRC changes by −0.008 for ESM-1b 650M, −0.038 for ESM-2 650M and +0.004 for ESM-2 3B; the ordering of the encoders and their margin over every baseline in Table 3 are unchanged.

### 3.2 Effect of test-set imbalance

The comparisons in §3.1 (Table 3) were carried out on the imbalanced test set. Here, we evaluate the same models on a balanced ($S_{i}$-Test-Bal, 1:1) and an imbalanced ($S_{i}$-Test, 1:383) test set to quantify the effect of class ratio on apparent performance. Table 4 reports both conditions for AcrNET(90%), AcrNET(40%), and AcrSeek (ESM-1b). The metrics—Accuracy, Precision, Recall, F1, and MCC—match those used in the AcrNET paper (Li *et al*. 2023), and per-fold oracle optimal thresholds are applied to remove threshold-selection effects. The oracle threshold makes absolute values optimistic, but it is applied uniformly to both conditions and therefore introduces no bias into the relative comparison.

**Table 4:** Performance on the balanced (1:1) and imbalanced (1:383) test sets.

Method	Class ratio	Acc	Precision	Recall	F1	MCC
AcrNET (90%)	Balanced (1:1)	0.8235 (0.0401)	0.7909 (0.0672)	0.8919 (0.0441)	0.8358 (0.0292)	0.6576 (0.0719)
	Imbalanced (1:383)	—^a^	0.4296 (0.2060)	0.2286 (0.0506)	0.2815 (0.0534)	0.3020 (0.0725)
AcrNET (40%)	Balanced (1:1)	0.7846 (0.0665)	0.7383 (0.0841)	0.9012 (0.0611)	0.8087 (0.0537)	0.5910 (0.1206)
	Imbalanced (1:383)	—^a^	0.0228 (0.0056)	0.5133 (0.0580)	0.0434 (0.0101)	0.0976 (0.0131)
AcrSeek (ESM-1b)	Balanced (1:1)	0.8190 (0.0312)	0.8510 (0.0849)	0.7883 (0.0655)	0.8136 (0.0253)	0.6463 (0.0658)
	Imbalanced (1:383)	—^a^	0.7657 (0.1328)	0.3276 (0.0172)	0.4563 (0.0325)	0.4984 (0.0488)

Per-fold oracle optimal thresholds are applied; values are 5-fold means with standard deviations in parentheses.

a Accuracy is omitted at the 1:383 ratio because predicting the majority class already gives 99.7% accuracy.

In the balanced setting AcrNET(90%) reaches F1 0.836 and MCC 0.658, but in the imbalanced setting it drops to F1 0.282 and MCC 0.302, with Precision falling from 0.791 to 0.430. This drop is driven by a 383-fold increase in negatives and the resulting surge of false positives, and it shows that high balanced-test performance does not reliably reflect operational performance. AcrNET(40%), with leakage controlled at 40% identity, attains a balanced F1 of 0.809—similar to AcrNET(90%)—but its imbalanced F1 of 0.043 is far below AcrNET(90%)’s 0.282. A substantial portion of AcrNET(90%)’s imbalanced performance therefore comes from allowing high-similarity sequences between training and test sets. AcrSeek (ESM-1b) reaches a balanced F1 of 0.814, matching AcrNET(90%), and its imbalanced F1 (0.456) and MCC (0.498) exceed both AcrNET variants (AcrNET(90%): F1 0.282, MCC 0.302; AcrNET(40%): F1 0.043, MCC 0.098). The balanced-to-imbalanced F1 drop is smallest for AcrSeek (−0.358) and larger for AcrNET(90%) (−0.554) and AcrNET(40%) (−0.766). Even AcrNET(90%)’s imbalanced F1 of 0.282 under the oracle threshold falls below the Fixed F1 range of 0.439–0.525 that AcrSeek achieves in Table 3 using inner-loop calibrated thresholds.

### 3.3 Ablation study

To isolate the contribution of each component of the combined loss, we fixed all other hyperparameters and varied only the loss-mixing weight λ. We compared focal-only (λ=0), combined (λ=0.5, the baseline), and triplet-only (λ=1) on ESM-2 650M embeddings; the triplet-only run was extended to 1300 epochs because of its slower convergence. Table 5 shows 5-fold results and Fig. 4 the corresponding learning curves. We then compare the design as a whole against a conventional classification head trained on the same representation.

**Figure 4:**
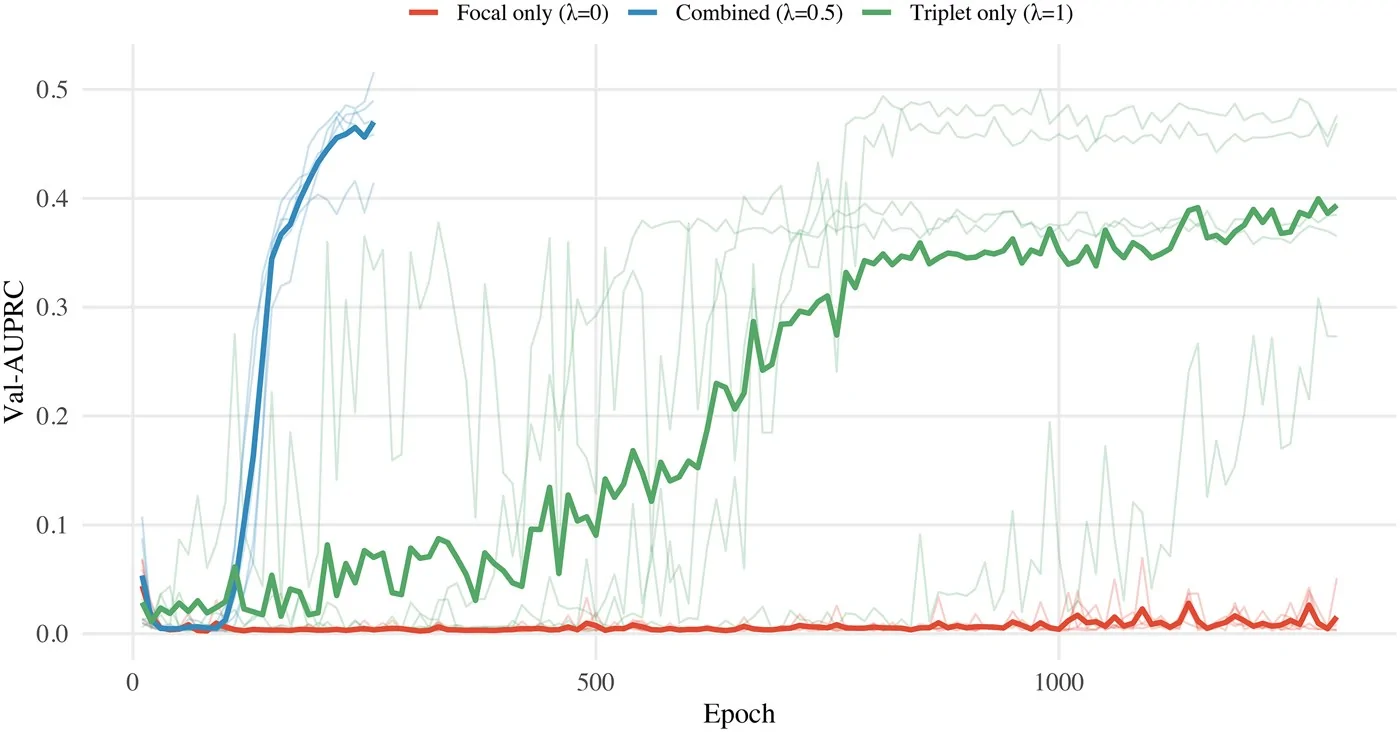
Validation AUPRC learning curves for the loss ablation. Solid lines are 5-fold means; semi-transparent lines show individual folds.

**Table 5:** Ablation of the loss composition, and comparison with a conventional classification head on the same projection head, for ESM-2 650M.

Setting	Epochs	AUPRC	AUROC	Prec@K	Oracle F1
Focal-only (λ=0)	1300	0.0138 (0.0133)	0.6606 (0.0436)	0.0257 (0.0279)	0.0444 (0.0336)
**Combined** (λ=0.5)	260	**0.4673** (0.0355)	0.9055 (0.0203)	**0.4914** (0.0314)	**0.5554** (0.0440)
Triplet-only (λ=1)	1300	0.4005 (0.0572)	0.8706 (0.0271)	0.4353 (0.0171)	0.4987 (0.0212)
Classification head,	280	0.4301 (0.0384)	**0.9203** (0.0144)	0.4352 (0.0256)	0.5178 (0.0244)
cross-entropy (1:383)					

Other hyperparameters of the combined loss are fixed and only λ is varied. The last row replaces the centroid-distance score of Equation 8 with a linear classification layer trained by cross-entropy on the full training set at its natural 1:383 ratio. Values are 5-fold means with standard deviations in parentheses. Bold values indicate the best value in each column. The bold row label "Combined" marks the configuration used as the baseline throughout the paper.

Triplet-only reaches AUPRC 0.401, 6.6 percentage points below the combined setting (0.467), yet on its own already produces clear positive clustering and positive–negative separation. Focal-only, by contrast, plateaus at AUPRC 0.014 and AUROC 0.661 even at 1300 epochs, indicating that a distance-based classification signal alone is not sufficient to structure the embedding space. The learning curves show that the combined loss converges at around 200 epochs while triplet-only converges at around 950 epochs—roughly a 4.7× difference in convergence epoch. These observations are consistent with a complementary role: the triplet loss shapes the geometry of the embedding space, and the focal loss accelerates that process through the centroid-distance classification signal.

Every setting above remains within a distance-based design. To ask whether a conventional classifier could have handled the imbalance instead, we compared one built on the same representation: a linear classification layer on the same projection head, trained by cross-entropy on the full training set at its natural 1:383 ratio, with neither a centroid nor a triplet term. Fixing the representation keeps the encoder and the model capacity out of the comparison, while scoring rule and objective differ jointly, so the contrast is between the two approaches rather than between individual components. The class weight was searched on the inner folds as for every other hyperparameter, and the unweighted setting was selected (Supplementary Section S6, available as supplementary data at *Bioinformatics* online). The combined loss leads on three of the four measures in Table 5: AUPRC 0.4673 against 0.4301, higher in all five folds, and Prec@K 0.4914 against 0.4352, while the classifier attains the higher AUROC, 0.9203 against 0.9055. AUROC integrates over all operating points, whereas AUPRC and Prec@K are dominated by the highest-ranked predictions, which at a 1:383 ratio constitute the only region from which candidates can realistically be drawn for experimental validation. The distance-based score therefore holds its advantage in the regime that governs candidate prioritisation, the task for which the model is intended. This comparison does not hinge on the learning rate the classifier inherited. Searching it jointly with the class weight selects $1\times 10^{-5}$ and raises the classifier to AUPRC 0.4305, a gain of 0.0004 whose paired 95% confidence interval across folds spans zero ([−0.018, +0.019]); the combined loss leads in all five folds under either configuration (Supplementary Section S6, available as supplementary data at *Bioinformatics* online).

### 3.4 Difficulty-stratified performance

We compare detection performance across the BLAST E-value-based difficulty groups defined in §2.9 (Fig. 5). AcrNET(90%) is excluded from this analysis because its loose leakage filter (allowing up to 90% identity) means that group-level differences could reflect leakage rather than model capability; AcRanker is also excluded because, as an off-the-shelf pretrained model, it provides no per-fold leakage control.

**Figure 5:**
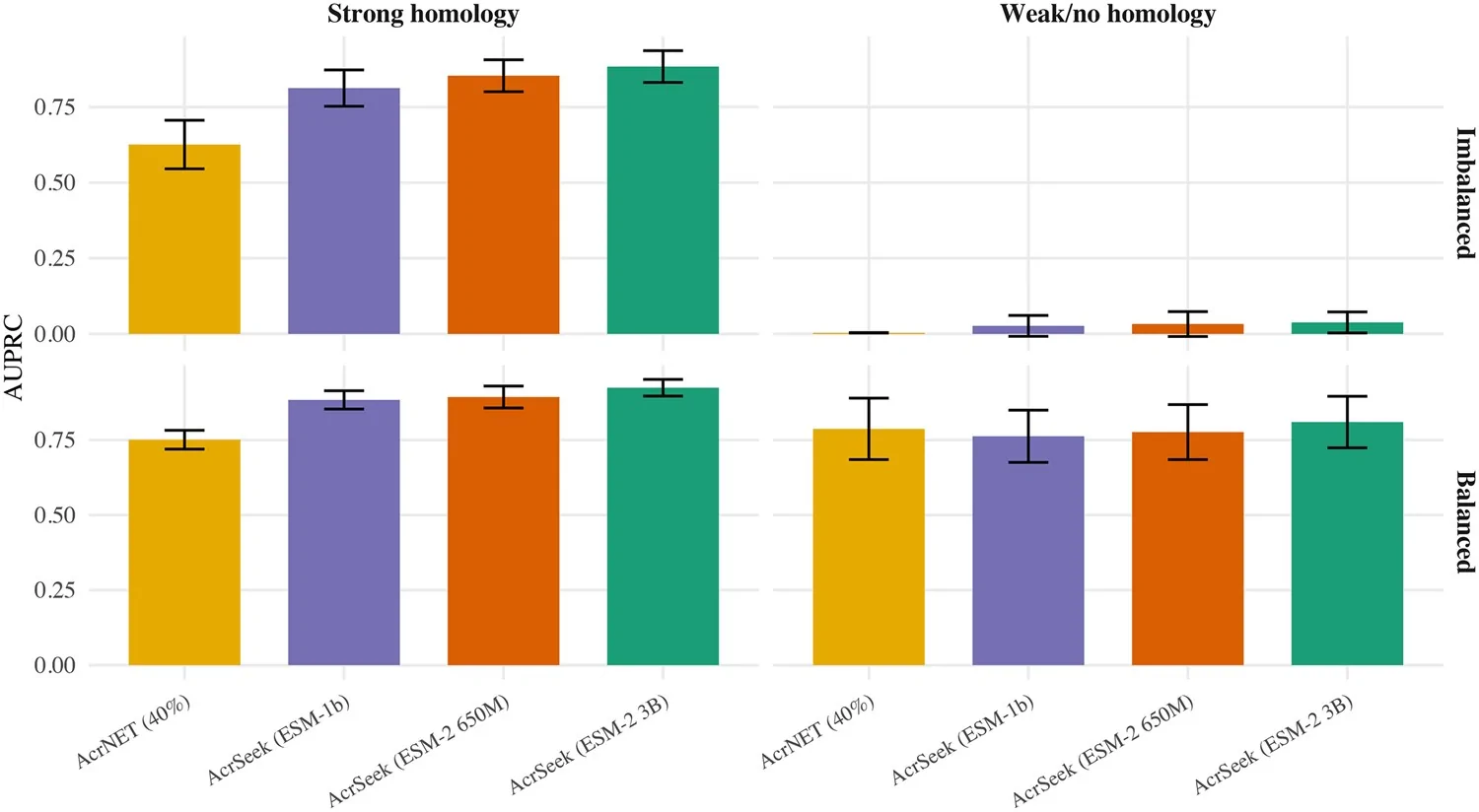
AUPRC by BLAST *E*-value-based difficulty group. Strong homology: *E*-value $<10^{-3}$; weak or no homology: *E*-value $\geq 10^{-3}$. Top row: imbalanced condition; bottom row: balanced condition. Bars are 5-fold means with standard-deviation error bars.

For the strong group (*E*-value $<10^{-3}$; 153 positives, 268 negatives, ≈ 1:1.75) under the imbalanced condition, AcrSeek (ESM-2 3B) reaches AUPRC 0.884—25.8 percentage points above AcrNET(40%, 0.626) and 18.7 percentage points above AcrSeek (ESM-1b, 0.813); under the balanced condition AcrSeek (ESM-2 3B, 0.924) likewise leads AcrNET(40%, 0.751) by 17.3 percentage points. The balanced-to-imbalanced AUPRC drop is also smaller for AcrSeek (ESM-2 3B, 3B, −4.0 percentage points) than for AcrNET(40%, −12.5 percentage points), and AUPRC increases consistently with PLM scale (ESM-1b 0.813 → ESM-2 650M 0.853 → ESM-2 3B 0.884). The class-ratio robustness observed in §3.2 therefore extends to the strong-homology subset.

In the weak group (*E*-value $\geq 10^{-3}$; 79 positives, 88 544 negatives, 1:1120), whose imbalance ratio is about three times that of the full test set, all models fall to AUPRC 0.004–0.038 under the imbalanced condition—below practical detection levels, with between-model differences inside the standard deviation—and converge to 0.762–0.810 under the balanced condition.

### 3.5 Performance across Acr families

Each P40 positive is held out in exactly one outer fold, so pooling the five folds gives one out-of-sample score for all 232 positives across 86 families. We decompose detection by Recall@*K*—the share of a family’s positives falling in the top *K* of their own fold, *K* being that fold’s positive count. Since *K* is shared by every family, the measure is independent of family size, whereas a per-family AUPRC would not be, its baseline scaling with the size of the group.

Detection varies over the full range. Among the 14 families contributing more than one sequence, Recall@*K* spans 0.000 to 1.000 under every encoder (Table S9, available as supplementary data at *Bioinformatics* online): AcrIIA8 (n=19) and AcrIIA7 (n=53) are recovered in almost every fold, whereas AcrIIC1 (n=7) is never recovered under any encoder and AcrIF6 (n=9) reaches at most 0.222. The same contrast appears at the coarser groupings by targeted CRISPR type and subtype: subtype II-A reaches Recall@*K* 0.62 to 0.76 across the encoders while II-C is never recovered, and type II leads type I throughout (Table S10, available as supplementary data at *Bioinformatics* online). Absence from training does not explain this—the family-stratified split (§2.1) leaves all 14 represented in every fold’s training set—and neither does family size, which correlates with Recall@*K* at ρ=0.21 and 0.23 (p>0.4) for ESM-1b and ESM-2 650M but ρ=0.72 (P=0.004) for ESM-2 3B. These correlations should not be over-read. Seven of the 14 families contribute two to four sequences, and at n=2 a Recall@*K* of exactly 0 or 1 arises by chance about half the time under the pooled detection rate. The scatter of the small families therefore attenuates any correlation with size rather than testing it, and among the seven largest families alone size and difficulty cannot be separated (ρ=0.70,P=0.078 for ESM-2 650M). We therefore do not read the spread as evidence either for or against a size effect, and turn instead to whether the ordering is specific to AcrSeek at all. Table 6 therefore sets the three encoders against AcrNET(40%), built on a different encoder, a cross-entropy objective and a softmax classifier with no centroid. It orders the families much as AcrSeek does (Spearman ρ=0.87, 0.82 and 0.56; all P<0.05) and likewise places AcrIIC1 last. Family difficulty is therefore a property of the families rather than of scoring by distance to a single centroid, though AcrSeek widens the range it produces (AUROC 0.439–1.000 against 0.638–0.976). The families that resist detection lie far from the centroid without forming a group of their own: under all three encoders their members are more dispersed among themselves than their family centroid is displaced from the global one (Table S13, available as supplementary data at *Bioinformatics* online).

**Table 6:** Per-family AUROC, pooled over the five outer folds, for families with more than one P40 member.

Family	*n*	**AcrSeek**			Reference
		ESM-1b	650M	3B	AcrNET
AcrIIA7	53	0.911	0.996	0.997	0.855
AcrID1	21	0.970	0.992	0.974	0.911
AcrIIA8	19	1.000	1.000	1.000	0.952
AcrIIA9	18	0.914	0.957	0.982	0.872
AcrIF11	14	0.977	0.997	0.999	0.946
AcrIF6	9	0.829	0.915	0.888	0.893
AcrIIC1	7	0.645	0.675	0.670	0.759
AcrIIA11	4	0.890	0.852	0.866	0.879
AcrIF9	4	0.812	0.861	0.988	0.804
AcrVIB	3	0.439	0.747	0.774	0.775
AcrIIA19	2	1.000	0.999	0.993	0.970
AcrIF8	2	0.813	0.808	0.503	0.928
AcrIIA2	2	0.999	0.999	0.995	0.976
AcrIIC2	2	0.615	0.599	0.585	0.638

The comparison uses AUROC because AcrNET’s positive-class probability saturates, placing most positives in a single tie block of up to 1640 sequences. Within that block the model supplies no ordering, so top-K membership cannot be attributed to a family. Per-family Recall@*K* is in Table S9, available as supplementary data at *Bioinformatics* online.

Seventy-two families contribute exactly one sequence, so holding it out leaves no member of its family in training; these positives estimate what the model does with an unseen family, and 70 of them also lack significant homology ($E\geq 10^{-3}$) to any training positive. Detection of these positives differs from that of the 160 whose families are represented in training. Pooled over the five folds, ESM-2 650M recovers 2 of the 72 (Recall@*K* 0.028 [0.01, 0.10]) against 112 of the 160 (0.700 [0.62, 0.77]; Table S11). Chance recovery at this depth is 0.003, so 0.028 stands about ninefold above it. At the head of the ranking they are strongly enriched: the top 500 candidates per fold (2.8% of 17 808 sequences) hold 19 to 23 of the 72 across the three encoders, against 2.0 expected by chance ($P<10^{-13},\;\mathrm{binomial}$).

### 3.6 Embedding space visualisation and mining dynamics

We analyse how training reshapes the embedding space using ESM-2 650M (Fold S1) (Fig. 6). In a UMAP projection of the raw 1280-dimensional ESM-2 embeddings, positives are scattered uniformly among negatives (Fig. 6A), whereas in the trained 256-dimensional projection they form a single tight cluster clearly separated from negatives (Fig. 6B).

**Figure 6:**
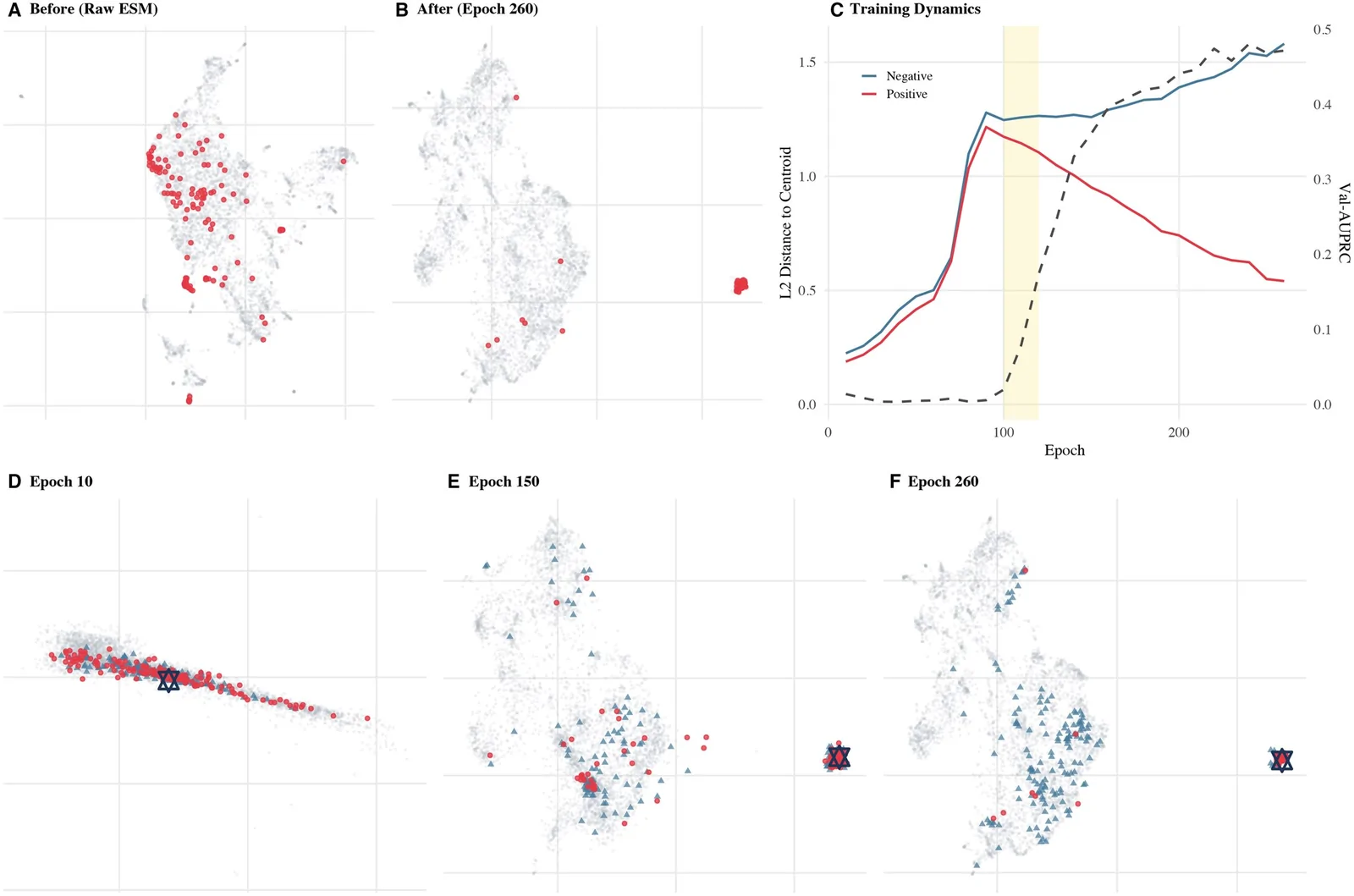
Reshaping of the embedding space by metric learning. (A) UMAP of the raw ESM-2 embeddings before training and (B) of the projected embeddings after training (red: positives; grey: a 5000-sample subset of negatives). (C) Mean L2 distance to the positive centroid (solid lines) and validation AUPRC (dashed line); the shaded region marks the phase-transition window (epochs 100–120). (D)–(F) Projected embeddings at epochs {10, 150, 260} (UMAP fitted on the final epoch); ▲: mining-pool members, ⋆: centroid.

The mean L2 distances from positives and negatives to the positive centroid (Fig. 6C) reveal a two-phase dynamic. During epochs 1–80, both distances grow together ($d_{\text{pos}}$: 0.19 → 1.04, $d_{\text{neg}}$: 0.22 → 1.10), indicating diffusion of embeddings onto the unit hypersphere. After epoch 80 the two trajectories cross like scissors: $d_{\text{pos}}$ decreases (1.04 → 0.54) while $d_{\text{neg}}$ increases (1.10 → 1.58), and the distance ratio $d_{\text{neg}}/d_{\text{pos}}$ rises from 1.06 at epoch 100 to 2.92 at epoch 260. Validation AUPRC stays below 0.02 up to epoch 100, then jumps to 0.08 at epoch 110 and 0.17 at epoch 120—coinciding with the moment when the distance ratio crosses approximately 1.1 (shaded region). This pattern is consistent with a phase transition in which the distance-based classifier only becomes effective once a critical level of separation has been reached.

The spatio-temporal dynamics of the offline mining pool (Fig. 6D–F) align with the same two phases. Early in training (epoch 10), the mining pool is dispersed throughout the embedding space, so a distance-based notion of hard negative is not yet well defined. By epoch 150 the pool concentrates along the boundary of the formed positive cluster, selecting only hard negatives near the centroid. The Jaccard distance between pool compositions across consecutive epochs shows the same transition: it stays at 0.85–0.96 during the embedding-expansion phase (epochs 40–80), indicating near-complete pool turnover from epoch to epoch, but stabilises at 0.34–0.57 after epoch 120. The mining-pool stabilisation phase (after epoch 120) coincides with the rapid validation AUPRC rise and the moment when the distance ratio exceeds 1.1, marking the onset of a regime in which the positive cluster supplies a consistent set of hard-negative candidates across epochs. This explains the slower convergence of triplet-only relative to the combined loss observed in §3.3: the focal loss is needed for the positive centroid—the reference point of the mining pool—to form quickly, which in turn stabilises the supply of hard negatives to the triplet loss earlier in training.

### 3.7 Residue-level interpretation: AcrIF11 case study

To assess whether the learned projection space distinguishes functional residues within a protein, we performed in-silico alanine scanning on a protein for which both a crystal structure and residue-level mutagenesis data are available.

We selected anti_CRISPR0490 (*Pseudomonas aeruginosa*, WP_038819808.1, AcrIF11 family, 132 aa), a protein that the model classified as positive with high confidence (passing the fixed threshold *P* ≥ 0.63 on the test set of its own fold) and for which residue-level mutagenesis data have been reported. Its predicted probability was *P* = 0.759, ranking 16 out of 17 810 sequences in the fold test set (top 0.09%), and we used the projection head from that fold for the residue analysis. Alanine was substituted at every position, with glycine used in place of alanine for native alanine residues; both wild-type and mutant sequences were processed through the inference procedure of §2.5, but with the raw L2 distance *d* to the positive centroid used in place of the sigmoid probability (Equation 8). The residue-level importance score was defined as $\Delta d_{i}=d_{\text{mut},i}-d_{\text{wt}}$. The relative perturbation magnitude against the within-protein background is also reported as a leave-one-out (LOO) robust *z*-score; full candidate-selection and computational details are in Supplementary Section S4.

Among the 132 residues, the H7 substitution gave the largest Δd=+0.380, about five times the value of the runner-up A28 (Δd=+0.082; Fig. 7). This corresponds to a LOO robust z=+14.62, far above the within-protein background distribution. H7 is one of the eight NAD-binding residues identified in crystallographic studies and occupies the histidine position of the conserved His-Tyr-Tyr-Glu (HYE) catalytic motif of bacterial ADP-ribosyltransferases (Niu *et al*. 2020). Hence the learned projection space produces a signal at a key active-site residue (H7) that is clearly distinguishable from the within-protein background.

**Figure 7:**
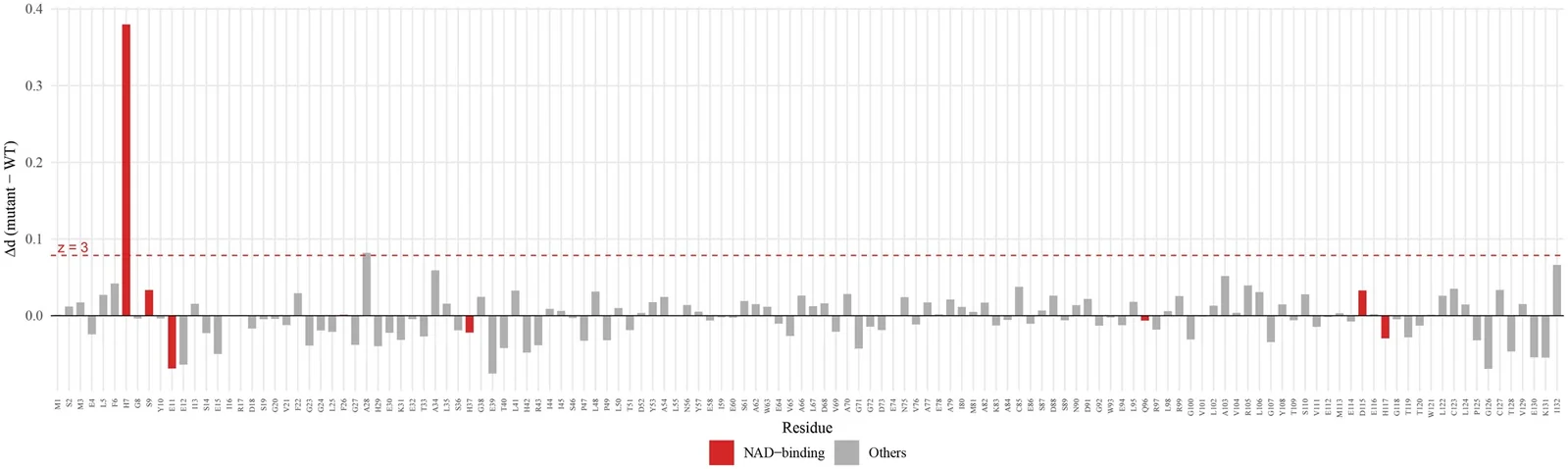
In-silico alanine scanning of AcrIF11 (anti_CRISPR0490). Profile of Δd (mutant − WT) over the 132 residues. Red bars: the eight NAD-binding residues (Niu *et al*. 2020); grey bars: the remaining 124 residues. The dashed red line marks the Δd level corresponding to robust z=3. H7 is detected as an extreme outlier (Δd=+0.380, robust z=+14.62, rank 1/132) and is the only NAD-binding residue that passes the threshold.

The remaining seven NAD-binding residues did not satisfy robust z≥ 3, and aggregate-level tests of whether all NAD-binding residues are concentrated near the top of the ranking (top-*K* hypergeometric and Wilcoxon rank-sum tests) failed to reject the null at significance level 0.05 (Supplementary Section S4, available as supplementary data at *Bioinformatics* online). The present result is therefore restricted to detecting a signal at the single residue H7. This nevertheless suggests that the projection space carries partial information about key functional residues, and consistent top-ranking of all eight NAD-binding residues is left for further validation.

The analysis is confined to a single protein because the requirements of the workflow—a high-confidence prediction, a structure of that same sequence, and published residue-level alanine-scanning mutagenesis—are jointly met by only one entry of the P40 positive set. Of the 91 sequences that pass the fixed threshold, only three are themselves present in the PDB at ≥ 95% identity, and of those only anti_CRISPR0490 has alanine-substitution data; the enumeration is given in Supplementary Section S4, available as supplementary data at *Bioinformatics* online.

## 4 Discussion

The primary finding of this work is that AcrSeek, a metric-learning approach, holds a structural performance advantage over the balanced-trained classification baselines under the approximately 1:383 class imbalance. Under matched 40% identity leakage control and a shared ESM-1b encoder (§3.1), AcrSeek (ESM-1b) exceeds AcrNET(40%) in AUPRC by about 21×, and the gap widens to about 30× with the ESM-2 3B encoder. Because this gap persists when both the PLM and the leakage condition are matched, the advantage is more consistent with differences in how the models were trained than with differences in the input representation.

The balanced-to-imbalanced (1:1–1:383) transition in §3.2 quantifies this structural difference. F1 collapses from 0.836 to 0.282 for AcrNET(90%) and from 0.809 to 0.043 for AcrNET(40%), whereas AcrSeek (ESM-1b) drops only from 0.814 to 0.456. Because per-fold oracle optimal thresholds are applied uniformly to all three models, the divergence reflects sensitivity to the class ratio itself rather than threshold-selection effects. The variable that separates the models here appears to be the ratio each was trained at rather than the choice between distance-based and classification-based scoring: both AcrNET variants are trained on balanced data and evaluated at 1:383, whereas AcrSeek and the classification head of §3.3 are trained at the deployment ratio and both perform substantially better under it than either AcrNET variant. The balanced training conventions of existing predictors are therefore a substantial part of what Table 3 reflects, and matching the training ratio to the deployment ratio emerges as a prerequisite for either approach.

In this Acr-detection setting, these results show that balanced-dataset evaluation systematically overestimates performance under imbalanced deployment conditions. Evaluation that does not reflect the positive density of the actual screening environment undermines the reliability of model comparisons, and the protocol used here—combining a 1:383 imbalance with 40% identity leakage control—directly addresses this limitation. The oracle-threshold results should nevertheless be read as an upper bound on threshold calibration; in deployment, AcrSeek’s Fixed F1, which uses inner-loop calibrated thresholds, is the more appropriate estimate of operational performance.

The ablation in §3.3 indicates that the two terms of the combined loss play complementary roles rather than acting as mutual regularisers. Triplet-only (λ=1) reaches AUPRC 0.401, showing that geometric structuring of the embedding space alone is sufficient for effective classification, whereas focal-only (λ=0) plateaus at 0.014, indicating that the distance-based classification signal cannot, on its own, induce structure in the embedding space. This asymmetry is consistent with the centroid-distance probability transformation in Equation 3 presupposing an already-structured embedding space: the triplet loss is responsible for the geometric work of positive aggregation and positive–negative separation, while the focal loss provides an auxiliary signal at the stage of reading the centroid distance as a probability.

The embedding-dynamics analysis in §3.6, illustrated on Fold S1, traces a trajectory consistent with this interpretation. As the centroid distance ratio $d_{\text{neg}}/d_{\text{pos}}$ crosses approximately 1.1 in the epoch 100–120 window, validation AUPRC jumps from below 0.02 to above 0.17—a pattern that reads as a phase transition in which the distance-based classifier begins to operate once a critical level of separation is reached. Over the same window, the Jaccard distance between consecutive offline mining pools stabilises from 0.85–0.96 to 0.34–0.57, marking the onset of a regime in which the positive cluster acts as a stable reference for the hard-negative candidate set. The roughly 4.7× convergence gap between combined (≈ 200 epochs) and triplet-only (≈ 950 epochs) follows naturally from this dynamic: the focal signal accelerates the formation of the positive centroid that anchors the mining pool, which in turn stabilises the supply of hard negatives to the triplet loss earlier in training. The two terms of the combined loss therefore divide labour across training phases, jointly contributing to embedding structuring and mining stabilisation.

The BLAST *E*-value-based difficulty stratification in §3.4 sharpens the practical scope of the framework. In the strong-homology group (*E*-value $<10^{-3}$), AcrSeek (ESM-2 3B) reaches AUPRC 0.884 even under the imbalanced condition, achieving a practical level of accuracy for prioritising candidates that bear remote but detectable sequence similarity to known Acrs. The balanced-to-imbalanced AUPRC drop is also smaller for AcrSeek (ESM-2 3B, −4.0 percentage points) than for AcrNET(40%, −12.5 percentage points), confirming that the class-ratio robustness observed in §3.2 extends consistently into the strong-homology subset.

In the weak- or no-homology group (*E*-value $\geq 10^{-3}$, ≈ 1:1120), by contrast, all models stay within AUPRC 0.004–0.038, well below practical detection levels, and the relative gap that AcrSeek maintains over AcrNET(40%) elsewhere narrows to within one standard deviation. This indicates that PLM embeddings and metric learning are effective mainly within the range where the distance structure to known positives can be exploited. The ceiling is not specific to distance-based scoring—AcrNET(40%), a softmax classifier with no centroid, falls to the same range—so at this similarity level it is a property of the detection problem rather than of the scoring rule. The framework is therefore better suited to prioritising candidates within a pool that retains at least weak homology to known Acrs than to discovering wholly novel families with negligible sequence similarity. §3.5 measures how far detection extends past that boundary: positives from unseen families, nearly all of them without detectable homology, are nevertheless enriched at the head of the ranking, and 26%–32% of them fall inside the top 500 candidates per fold (Table S12, available as supplementary data at *Bioinformatics* online).

In the AcrIF11 case study (§3.7), the learned projection space produced an extreme signal at H7 (Δd=+0.380, robust z=+14.62, rank 1/132) among the eight known NAD-binding residues. H7 corresponds to the histidine position of the conserved HYE motif of bacterial ADP-ribosyltransferases (Niu *et al*. 2020), and its perturbation is extreme relative to the within-protein background distribution (robust z=+14.62), suggesting that the projection space retains a partial capacity to distinguish key functional residues from the rest of the protein.

However, H7 was the only one of the eight NAD-binding residues to satisfy robust z≥3, and aggregate-level tests (top-*K* hypergeometric and Wilcoxon rank-sum) failed to reject the null at significance level 0.05 (Supplementary Section S4, available as supplementary data at *Bioinformatics* online). The result is therefore confined to detecting a signal at a single residue and does not establish the projection space as a general interpretative tool that consistently ranks the full set of functional residues at the top. Several factors plausibly contribute to this partial behaviour: (i) under mean-pooled embeddings, the effect of any individual residue perturbation on the pooled representation is systematically diluted by the averaging operation; (ii) the impact of an alanine substitution is not homogeneous across residue types, and at hydrophobic positions side-chain replacement may induce relatively small local conformational changes; and (iii) the opposing signs of Δd at H7 and E11 indicate that individual-residue contributions depend strongly on structural context even within the same NAD-binding function. Developing the projection space into a general residue-level prioritisation tool will likely require both per-residue attention-based attribution (Abnar and Zuidema 2020, Vig *et al*. 2021, Achtibat *et al*. 2024) in place of mean pooling, and PLM fine-tuning to strengthen residue-level discriminability.

Four limitations frame this study. First, all results depend on the representation quality of the frozen PLM, and discriminative signals not already captured in the embeddings are difficult to recover without fine-tuning; the monotonic performance gain with PLM scale (§3.1) suggests room for further fine-tuning-based improvement, but realising it would require training strategies that control overfitting under a small positive set. Second, the size of the positive set (P40, 232 sequences) limits how completely the generalisation boundary of the embedding space can be probed experimentally, and the limitation observed in the weak-homology group may partly reflect this constraint. The per-family decomposition (§3.5) also bounds what a single positive centroid can be asked to represent, and indicates that adding a second prototype at a family mean would not obviously recover the families AcrSeek misses. Third, the study is restricted to computational evaluation, and the novel candidates ranked at the top by the model have not been validated experimentally. Fourth, the negatives are prophage-derived candidates rather than experimentally confirmed non-Acrs, and no verified non-Acr set exists at this scale. Removing the 157 sequences sharing ≥ 40% identity with an anti-CRISPRdb entry (§2.1) bounds the most obvious contamination but cannot exclude undiscovered Acrs, and the offline mining stage selects precisely the negatives nearest the positive centroid, where such sequences would sit; the semi-hard condition of Equation 7 excludes them from the triplet term, but the focal term supervises every mined negative. Any negative that is in fact an Acr is therefore scored as a false positive, so the precision figures we report are lower bounds, and hard-negative mining sharpens the decision boundary at the cost of pressure on the region where undiscovered families would lie. Treating the negatives as unlabelled rather than negative, as in positive–unlabelled learning, could relax this trade-off.

These limitations point to natural directions for follow-up work. The AUPRC of approximately 0.88 in the strong-homology group (§3.4) provides a practical basis for selecting candidates for experimental triage, and *in vitro* or *in vivo* validation of top-ranked novel Acr candidates is the most immediate next step. Because none of the components of the pipeline—PLM embeddings, metric learning, hybrid negative mining, and the imbalanced evaluation protocol—relies on Acr-specific assumptions, extending the approach to other protein-function detection problems with scarce positive samples is a natural follow-up that would test the generality of the framework. Finally, advancing the residue-level analysis from a single case study to a systematic assessment will require both the introduction of per-residue attribution and quantitative validation on proteins with available mutagenesis data.

## 5 Conclusion

We presented AcrSeek, a metric-learning framework that combines a frozen PLM encoder, a joint triplet–focal objective, and hybrid negative mining, together with an evaluation protocol that controls leakage through 40% identity clustering and assesses Acr detection at the extreme class ratio of approximately 1:383. On the imbalanced test set, AcrSeek (ESM-2 3B) exceeded AcrNET in AUPRC by about 30× under matched leakage conditions, and reached a practical level of performance (AUPRC ∼ 0.88) in the strong-homology group. These results indicate that metric learning remains structurally robust under extreme class imbalance and establish AcrSeek as a practical tool for large-scale prioritisation of candidates that retain detectable homology to known Acrs. Because none of the framework’s components rely on Acr-specific assumptions, the same approach may also serve other protein-function detection problems in which positive examples are scarce.

## Supplementary Material

btag729_Supplementary_Data

## Data Availability

The processed datasets, ESM-1b/ESM-2 embeddings, and trained model checkpoints are deposited at Zenodo (DOI: 10.5281/zenodo.20115442). Source code, environment, and reproduction workflows are freely available on GitHub at https://github.com/jeongchans/acrseek, and the release used for this study is archived at Zenodo (DOI: 10.5281/zenodo.21946803).
