# Supplementary material for: AcrSeek: metric learning with hybrid negative mining for anti-CRISPR protein detection under extreme class imbalance
Source: Bioinformatics. 2026 Sep 29;42(10):btag729. doi: 10.1093/bioinformatics/btag729 (PMC13657427; doi:10.1093/bioinformatics/btag729)
Supplement: Supplementary file 1 [file btag729_supplementary_data.pdf]

Supplementary Material for "AcrSeek: Metric Learning with Hybrid Negative Mining for Anti-CRISPR Protein Detection under Extreme Class Imbalance"

S1 Hyperparameter search space

This section provides further detail on the inner-loop grid search summarised in §2.6 of the main paper.

S1.1 Search space and grid size

We searched over four hyperparameters (HPs): the learning rate η, the projection dimension d, the loss-mixing weight λ, and the triplet margin m. Per-embedding search ranges are listed in Table S1. Each grid contained $3 \times 3 \times 3 \times 2 = 54$ combinations, and each combination was evaluated on five inner folds, giving 270 training runs per embedding.

Table S1: Per-embedding hyperparameter search ranges. The grid contains $3 \times 3 \times 3 \times 2 = 54$ combinations per embedding.

**Setting**	**Symbol**	**ESM-1b 650M**	**ESM-2 650M**	**ESM-2 3B**
Learning rate	η	2e-5, 3e-5, 5e-5	3e-5, 5e-5, 1e-4	2e-5, 3e-5, 5e-5
Projection dim.	d	64, 128, 256	64, 128, 256	128, 256, 512
Loss weight	λ	0.3, 0.5, 0.7	0.3, 0.5, 0.7	0.3, 0.5, 0.7
Margin	m	0.5, 1.0	0.5, 1.0	0.5, 1.0
Epoch candidates	E	$\{10, 20, \ldots, 1{,}000\}$ (100 candidates)		

The remaining training settings (optimiser, weight decay, batch size, etc.) were held constant across all embeddings and combinations and are listed in the lower part of Table 2 of the main paper.

S1.2 Epoch selection and smoothing

Each combination was trained for up to $E_{\max} = 1{,}000$ epochs, with S_i-Val-Sub performance recorded at 100 candidate points spaced 10 epochs apart. The optimal epoch was the candidate with the highest validation area under the precision–recall curve (AUPRC) after smoothing with a centred moving average of window size $w = 5$. The smoothing operator is a simple moving average that shrinks at the boundaries:

$$\hat{y}_i = \frac{1}{|\mathcal{W}_i|} \sum_{j \in \mathcal{W}_i} y_j, \quad \mathcal{W}_i = \{j : \max(0,\, i - \lfloor w/2 \rfloor) \le j < \min(n,\, i + \lfloor w/2 \rfloor + 1)\}, \tag{S1}$$

where y_j is the raw validation AUPRC at the j-th candidate point. With only a few tens of validation positives per fold, PR curves become step-like and AUPRC estimates have high variance; smoothing mitigates this.

S1.3 Shared HP strategy

Standard nested cross-validation lets each outer fold choose its own HPs from the inner loop (the fold-independent strategy). In our setting, however, each inner-loop fold contains only about 37 validation positives, so AUPRC estimates are noisy. Selecting the per-fold maximum among 54 candidates under such noise is vulnerable to the winner's curse: a combination overestimated by validation noise tends to be selected.

To mitigate this risk, we adopted a Shared HP strategy — a deliberate modification of standard nested cross-validation — with the following procedure:

1. For each of the 54 HP combinations, we computed the mean smoothed validation AUPRC across the five inner folds.
2. We selected the combination with the highest mean as the representative HP for the embedding. Averaging reduces per-fold noise and yields a more stable choice than per-fold selection.
3. We set the final epoch to the median of the per-fold optimal epochs for the selected HP.
4. We applied this single (HP, epoch) configuration to every outer-loop fold.

The selected hyperparameters are listed in the upper part of Table 2 of the main paper.

Averaging over the five inner folds in step 1 couples HP selection across outer folds: the configuration applied to S_i is chosen using validation scores from the other four inner loops, whose validation sets are drawn from the remaining folds and therefore intersect S_i-Test. Under fold-independent selection each outer fold draws on its own inner loop alone, and this coupling does not arise.

The coupling is confined to the selection step. No model is trained or early-stopped on S_i-Test, and the outer estimate for S_i comes from a model fitted only to S_i-Train-Sub. Each contributing inner fold evaluates a different model fitted to a different training set, so a validation score measured on sequences that also occur in S_i-Test does not characterise the model subsequently evaluated on them. What is shared across folds is a single discrete choice among the 54 (HP, epoch) combinations, taken on a five-fold average. Whether this coupling inflates the outer estimate is an empirical question, addressed in §S1.4.

S1.4 Empirical comparison with the fold-independent strategy

We examined this question empirically by re-running the outer loop under fold-independent selection for all three encoders on the same 5-fold splits. Each fold was trained with its own inner-loop best (HP, epoch) configuration. Table S2 reports the outcome.

Table S2: Shared HP versus fold-independent HP selection. Test AUPRC is the 5-fold mean (standard deviation); Δ is the paired mean difference (fold-independent − Shared HP). The val–test gap is the difference between the mean inner-validation AUPRC obtained under each selection rule and the corresponding test AUPRC.

	**Test AUPRC**			**Val–test gap**	
**Encoder**	**Shared HP**	**Fold-indep.**	Δ	**Shared HP**	**Fold-indep.**
ESM-1b 650M	0.3547 (0.0524)	0.3465 (0.0546)	−0.0082	0.0107	0.0410
ESM-2 650M	0.4673 (0.0355)	0.4291 (0.0478)	−0.0383	0.0017	0.0616
ESM-2 3B	0.5077 (0.0523)	0.5119 (0.0575)	+0.0043	0.0221	0.0331

The difference Δ is not of consistent sign across encoders: fold-independent selection gives a lower test AUPRC for ESM-1b 650M and ESM-2 650M, and a marginally higher one for ESM-2 3B. For ESM-2 650M the difference is negative in all five folds; for the other two encoders

it changes sign between folds. Coupling at the selection stage would be expected to shift the Shared HP estimate in one direction, and no such common direction is present across the three encoders.

The asymmetry between inner-validation and test AUPRC replicates across all three encoders. Under Shared HP the inner-validation estimate tracks the test value closely (gaps of 0.011, 0.002 and 0.022); under fold-independent selection the gap is larger in every encoder (0.041, 0.062 and 0.033). This is consistent with the winner's curse incurred by taking a per-fold maximum over 54 candidates when only ~37 validation positives are available. The fold-independent estimate therefore carries a selection bias of its own, in each case larger than the corresponding gap under Shared HP.

Both mechanisms displace Δ in the same direction: selection coupling by raising the Shared HP estimate, the winner's curse by lowering the fold-independent one. The observed difference is therefore their combined effect and cannot be attributed to either alone, but it does place an upper bound on each, the largest across the three encoders being 0.038 (ESM-2 650M). Applying that bound leaves the conclusions of §3.1 of the main paper unchanged: under fold-independent selection the three encoders reach 0.347, 0.429 and 0.512, preserving both their ordering and their margin over every baseline in Table 3.

S1.5 Hyperparameters for ablations

For the loss-function ablation in §3.3 of the main paper, we fixed the HPs optimised for the combined loss and varied only λ (focal-only: $\lambda = 0$; triplet-only: $\lambda = 1$); no separate hyperparameter search was performed.

S2 Per-fold detailed results

Figure S1 complements the 5-fold means in Table 3 of the main paper by reporting individual values for every fold (S_1–S_5). The figure shows the per-fold variance pattern of all six methods across the four metrics (AUPRC, AUROC, Prec@K, FPR@95). AcrNET(40%) FPR@95 values vary widely across folds and arise from a tie band at the noise floor of its score distribution (footnote § of Table 3 of the main paper); these values are unstable and should be interpreted with caution.

S3 AcrNET reproduction details

This section supplements the AcrNET reproduction described in §2.7 of the main paper. Figure S2 illustrates the construction of the balanced training datasets (S_i-Train-Bal and S_i-Train-Sub-Bal) used for AcrNET comparison.

S3.1 Reimplementation scope and model architecture

The model architecture and training-dataset composition are described in §2.7 of the main paper. We reimplemented the model using the original code (GitHub: `banma12956/AcrNET`) as a reference and verified agreement on architecture, training loop, and hyperparameters. At inference, the softmax positive-class probability was used as the score; no probability calibration was applied, and the binary classification threshold was 0.5.

The five S_i-Train-Bal sets together contained 1,741 unique sequences after deduplication, for which ESM-1b 650M embeddings were computed. For hyperparameter validation we split S_i-Train-Bal 80/20 into S_i-Train-Bal-Sub (training) and S_i-Val-Bal-Sub (validation). Each S_i-Train-Sub-Bal contained approximately 148 positives and 148 negatives per fold.

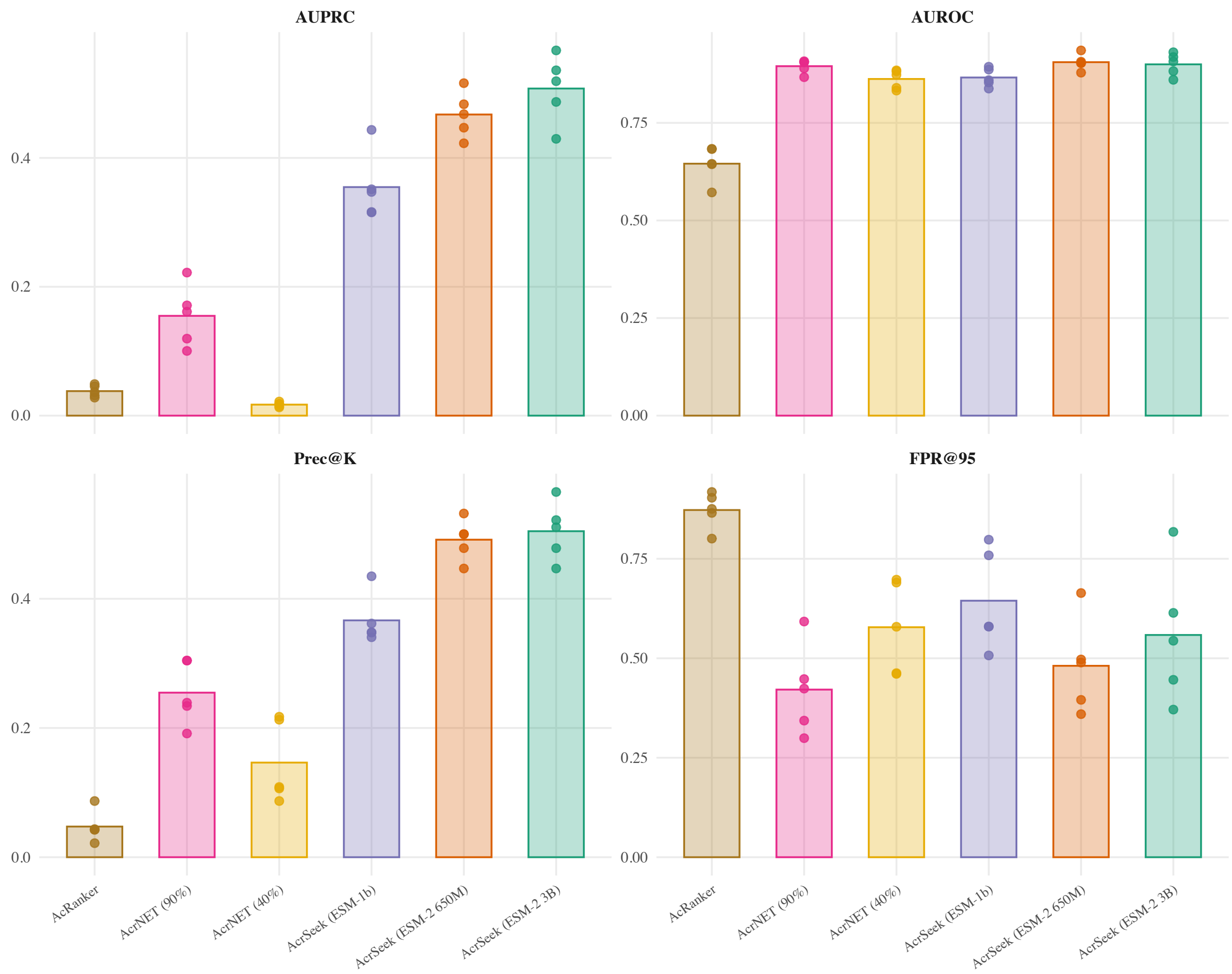

Figure S1: Per-fold values of the main metrics on the imbalanced test set. Points show individual folds (S_1–S_5) and bars show 5-fold means. Higher is better for AUPRC, AUROC, and Prec@K; lower is better for FPR@95.

S3.2 Hyperparameter validation

To verify that the hyperparameters reported in the original paper (Li et al., 2023) remain optimal in our reimplementation, we performed a grid search using the same grid reported in the original paper's appendix. The grid was steps $\in \{30, 300, 3{,}000\}$ $\times$ lr $\in \{0.1, 0.01, 0.001, 0.0001\}$ (12 combinations), with batch size fixed at 16 and the optimum selected by 5-fold mean accuracy.

AcrNET(90%) track. The published HP (steps = 3,000, lr = 0.001) achieved a validation accuracy of 0.9415 and was confirmed as optimal (Table S3).

Table S3: AcrNET(90%) HP grid-search results (5-fold mean accuracy on S_i-Val-Bal-Sub). Bold indicates the optimal combination.

Steps	lr=0.1	lr=0.01	lr=0.001	lr=0.0001
30	0.8086	0.9154	0.9068	0.8858
300	0.7692	0.9312	0.9264	0.9160
3,000	0.7327	0.9402	**0.9415**	0.9396

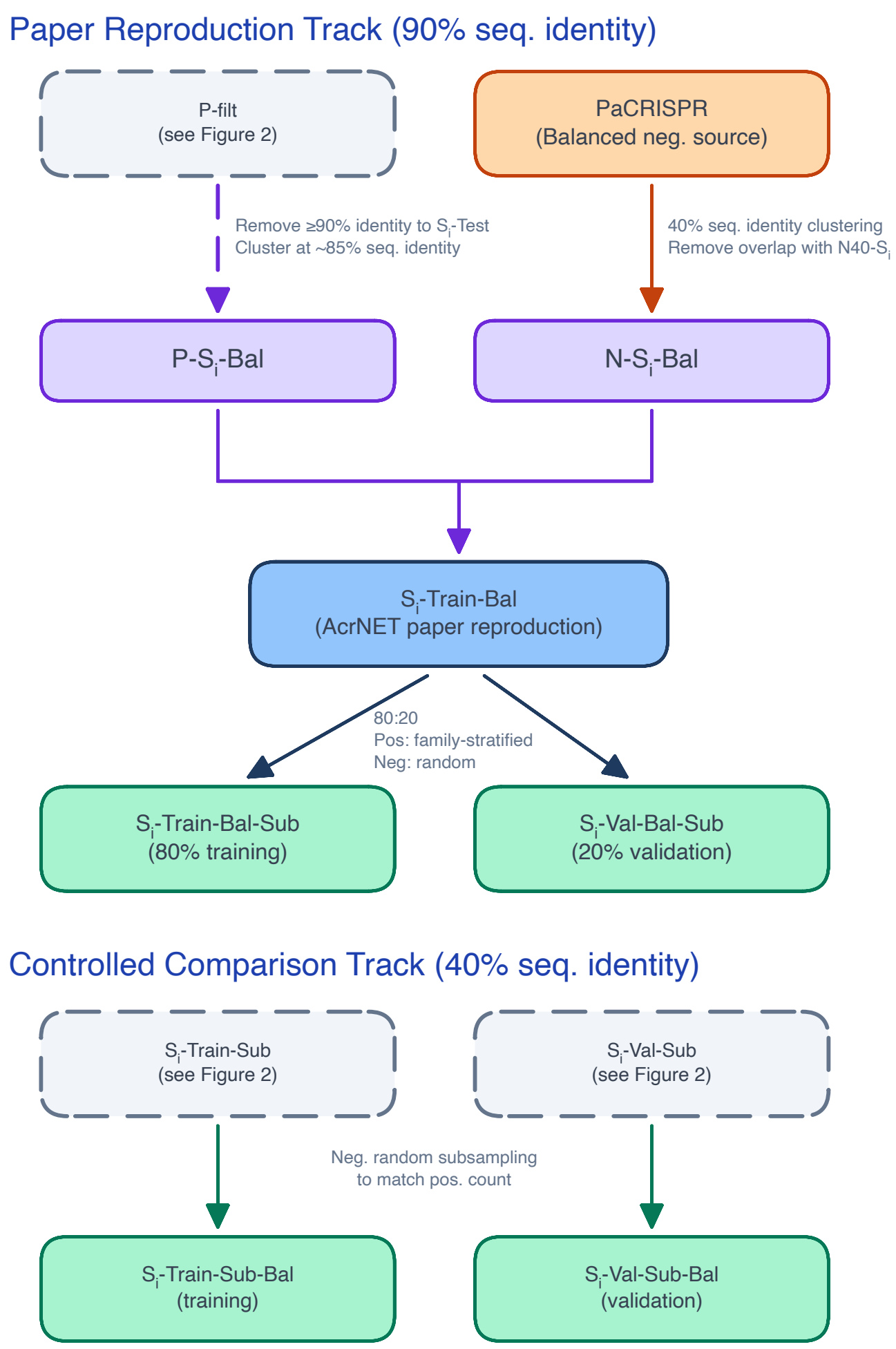

Figure S2: Construction of the balanced training sets used for AcrNET comparison. The **paper-reproduction track (S_i-Train-Bal)** is derived from PaCRISPR and P-filt and permits up to 90% train–test positive between-similarity. The **controlled-comparison track (S_i-Train-Sub-Bal)** is constructed from S_i-Train-Sub by negative subsampling, with train–test positive between-similarity capped at 40%. See §2.7 of the main paper for the detailed construction.

AcrNET(40%) track. Repeating the same grid search on S_i-Train-Sub-Bal / S_i-Val-Sub-Bal selected steps = 3,000, lr = 0.01 (validation accuracy 0.8081 ± 0.0335) as optimal. Unlike the published setting (lr = 0.001), the search selected a learning rate one level higher, possibly reflecting the smaller training set produced by 40% identity filtering.

S3.3 Reproduction verification

Table S4 reports the metrics obtained when training on S_i-Train-Bal-Sub and evaluating on S_i-Val-Bal-Sub with the published HP (steps = 3,000, lr = 0.001). On every metric, our 5-fold mean is comparable to or marginally above the values reported for the original paper's Transformer-only branch (Li et al., 2023). Because the original paper reports only single values without standard deviations, an exact statistical comparison is not feasible; the 5-fold mean nevertheless supports the fidelity of the reproduction across metrics.

Table S4: AcrNET reproduction verification. 5-fold mean (standard deviation in parentheses) on S_i-Val-Bal-Sub. Values for the original paper (Li et al., 2023) are taken from the Transformer-only branch (single value, no standard deviation reported).

**Metric**	**AcrNET (original)**	**AcrNET (reproduced)**
Acc	0.9326	0.9415 (0.0091)
Precision	0.9351	0.9402 (0.0287)
Recall	0.9248	0.9449 (0.0315)
F1	0.9299	0.9416 (0.0092)
MCC	0.8652	0.8846 (0.0167)

S4 In-silico alanine scanning details (AcrIF11)

This section supplements the case study of §3.7 of the main paper with details on candidate selection, protocol, full ranking, statistical tests, and threshold sensitivity.

S4.1 Candidate selection

A candidate for residue-level analysis had to satisfy three conditions: (i) the sequence had to be classified as positive in the test set of its assigned fold by passing the fixed threshold $p \geq 0.63$; (ii) a structure of that same sequence had to be available, so that the residues carrying the signal could be placed in a structural context and the literature numbering could be verified against the native numbering; and (iii) residue-level alanine-scanning mutagenesis had to have been reported for that sequence. Condition (i) is a precondition rather than a convenience: the residue-level score Δd measures displacement away from the positive centroid, so it is interpretable only for a sequence the model already places inside the positive region. Condition (ii) was assessed by querying each threshold-passing sequence with `blastp` against the protein SEQRES records of the wwPDB (accessed 2026-08-04); a sequence was counted as having its own structure when the best hit reached at least 95% identity over at least 90% of the query.

Applying condition (i) to the Acr-positive set of the P40 dataset retained 91 sequences spanning six families. Of these, only three sequences satisfy condition (ii): anti_CRISPR0490 (AcrIF11; 6KYF chain A, 100% identity over 100% of the query), anti_CRISPR0475 (AcrID1; 2H36 chain X, 100% over 99%), and anti_CRISPR3672 (AcrIC5; 7YHR chain A, 100% over 100%). The remaining 88 sequences reach at most 68.8% identity to any PDB chain, and seven have no hit at all. Condition (iii) is met by one of the three, anti_CRISPR0490, whose eight NAD-binding residues were experimentally validated by alanine substitution (Niu et al., 2020); it was therefore the analysis target. Table S5 enumerates the six families.

S4.2 Protocol and metrics

For each position in the target sequence, we generated a mutant by substituting the residue with alanine; where the wild-type residue was already alanine, glycine was used instead, ensuring a consistent side-chain perturbation at every position. We applied the inference procedure of §2.5 of the main paper to each mutant and to the wild-type sequence. The mean-pooled ESM-2 embedding $\mathbf{x} \in \mathbb{R}^{1280}$ was projected to $\mathbb{R}^{256}$ by the trained projection head, and the L2 distance

Table S5: Enumeration of the six Acr families passing the fixed threshold $p \geq 0.63$ (condition (i)). n is the number of threshold-passing sequences and $\max p$ the highest predicted probability within the family. "Own structure" counts sequences whose best `blastp` hit against wwPDB SEQRES reaches $\geq$ 95% identity over $\geq$ 90% of the query (condition (ii)); "best identity" is the range of best-hit identities over the remaining members of the family. Condition (iii) was assessed only for the families with at least one own structure; "—" marks families excluded at condition (ii).

**Family**	n	$\max p$	**Own structure**	**Best identity**	**Ala-scan data**
AcrIIA7	47	0.978	0 / 47	27.2–68.8%	—
AcrIIA8	17	0.958	0 / 17	25.2–42.9%	—
AcrIIA9	11	0.956	0 / 11	25.0–38.2%*	—
AcrIF11	10	0.913	1 / 10	25.0–41.3%	yes
AcrID1	5	0.756	1 / 5	27.1–58.3%	no
AcrIC5	1	0.727	1 / 1	—	no

*Seven of the eleven AcrIIA9 sequences produced no `blastp` hit against the PDB at all.

to the positive centroid $\boldsymbol{\mu}$ was computed as $d = \|\mathbf{z} - \boldsymbol{\mu}\|_2$. The importance score for residue i is the difference in distance:

$$\Delta d_i = d_{\text{mut},i} - d_{\text{wt}}. \tag{S2}$$

The wild-type distance was $d_{\text{wt}} = 0.7553$, and the predicted probability $p = 0.759$ produced by the inference procedure follows from this distance via the sigmoid transformation in Eq. 8 of the main paper.

For residue analysis we used the raw L2 distance rather than the sigmoid probability used at inference (Eq. 8 of the main paper). For this high-confidence positive case, $\hat{p}$ approaches the saturation region of the sigmoid, where residue-level distance changes are nonlinearly compressed in probability space; the raw distance reflects the geometric displacement in the projection space directly on a linear scale and therefore preserves residue-level resolution.

To compare residues within the protein, we computed a leave-one-out (LOO) robust z-score over the Δd distribution as an auxiliary indicator. The LOO robust z for residue i is

$$z_i^{\text{LOO}} = \frac{\Delta d_i - \text{median}(\Delta d_{-i})}{1.4826 \cdot \text{MAD}(\Delta d_{-i})}, \tag{S3}$$

where Δd_{-i} is the Δd vector with the i-th component removed and MAD denotes the median absolute deviation. The constant 1.4826 rescales MAD so that $\sigma \approx 1.4826 \cdot \text{MAD}$ holds under a normal assumption. The leave-one-out construction prevents a single extreme value from inflating its own scale. Because the Δd distribution is heavy-tailed, we did not convert robust z to normal-approximation p-values; we used it only as a descriptive measure of the perturbation magnitude relative to the background distribution across the protein's residues.

S4.3 Full ranking of NAD-binding residues

The eight NAD-binding residues of AcrIF11 reported by Niu et al. (2020) are listed with their Δd ranks in Table S6, and their structural locations are shown in Figure S3. H7 stands out as a single extreme value at rank 1, whereas the remaining seven residues are distributed widely across the ranking. S9 (rank 11) and D115 (rank 12) lie in the upper tail but do not meet the $z \geq 3$ criterion; F26 (rank 62), Q96 (rank 84), H37, and H117 fall in the middle to lower part of the ranking; and E11 (rank 130) shows a perturbation in the opposite direction. D115 corresponds structurally to the conserved catalytic Glu of the HYE motif found in bacterial ADP-ribosyltransferases and is a critical catalytic residue whose single substitution is sufficient to abolish ADP-ribosylation activity (Niu et al., 2020).

Table S6: Δd and robust-z ranks for the eight NAD-binding residues of AcrIF11. Rank denotes the position in descending Δd order across the 132 residues. NAD interactions are taken from Niu et al. (2020). D115 corresponds to the catalytic Glu of the HYE motif and is a critical catalytic residue.

Residue	NAD interaction	Δd	Robust z	Rank
H7	Hydrogen bond (ADP ribose)	+0.380	+14.62	1
S9	Hydrogen bond (ADP ribose)	+0.033	+1.29	11
D115	Hydrogen bond (catalytic)	+0.033	+1.27	12
F26	Hydrophobic	+0.001	+0.04	62
Q96	Hydrogen bond	−0.006	−0.25	84
H37	π-π (nicotinamide)	−0.022	−0.87	104
H117	Hydrophobic	−0.029	−1.17	111
E11	Hydrogen bond (ADP ribose)	−0.069	−2.70	130

S4.4 Aggregate-level statistical tests

To assess whether the eight NAD-binding residues concentrate among the top K entries of the Δd ranking, we performed a one-tailed hypergeometric top-K enrichment test ($n = 132$, $m = 8$, $K \in \{10, 20, 30\}$). We additionally compared the Δd distribution of the eight NAD-binding residues directly against that of the 124 non-binding residues using a one-tailed Wilcoxon rank-sum test (alternative hypothesis: NAD-binding > others; Table S7).

The hypergeometric p-values were 0.477 at $K = 10$, 0.101 at $K = 20$, and 0.262 at $K = 30$, none reaching the 0.05 significance level. The Wilcoxon test was likewise not significant ($p = 0.437$). The median Δd for NAD-binding residues (−0.003) was lower than the median for non-binding residues (+0.000), so no distributional dominance is observed. At the aggregate level, the NAD-binding residues as a set do not appear to concentrate systematically in the upper tail of Δd.

Table S7: Aggregate-level statistical tests for the NAD-binding residues of AcrIF11. $n = 132$ is the total number of residues and $m = 8$ is the number of NAD-binding residues. Hypergeometric reports the one-tailed p-value of NAD-binding overlap within the top K of Δd; Wilcoxon rank-sum reports the one-tailed p-value for the alternative hypothesis that NAD-binding residues exceed non-binding residues.

Test	Parameter	Observation	p-value
Hypergeometric	$K = 10$	overlap = 1	0.477
	$K = 20$	overlap = 3	0.101
	$K = 30$	overlap = 3	0.262
Wilcoxon rank-sum	NAD > others	$\text{median}_{\text{NAD}} = -0.003$<br>$\text{median}_{\text{other}} = +0.000$	0.437

S4.5 Sensitivity of the robust-z threshold

The threshold $z \geq 3$ used as the single-outlier criterion follows the conventional 3-sigma rule. To assess the impact of this choice, we lowered the threshold across $z \in \{3.0, 2.5, 2.0\}$ and recorded the residues flagged at each setting and their overlap with NAD-binding residues (Table S8). At $z \geq 3.0$, H7 was flagged together with A28 ($z = +3.15$), which is not an NAD-binding residue. Lowering the threshold to $z \geq 2.5$ added I132 ($z = +2.55$), and lowering it further to $z \geq 2.0$ also included A34 ($z = +2.28$), reducing precision to 1/4. Relaxing the threshold therefore

admitted more non-NAD-binding positions, but H7's $z = +14.62$ remained the strongest signal in this analysis, separated from the next candidate ($z = +3.15$) by approximately 11 robust-z units.

Table S8: Sensitivity of the robust-z threshold. Flagged is the number of residues with z_i^{LOO} at or above the threshold; NAD overlap is the number of NAD-binding residues among them; Precision is NAD overlap divided by Flagged.

z threshold	Flagged	NAD overlap	Precision	Flagged residues
3.0	2	1	0.50	H7, A28
2.5	3	1	0.33	H7, A28, I132
2.0	4	1	0.25	H7, A28, I132, A34

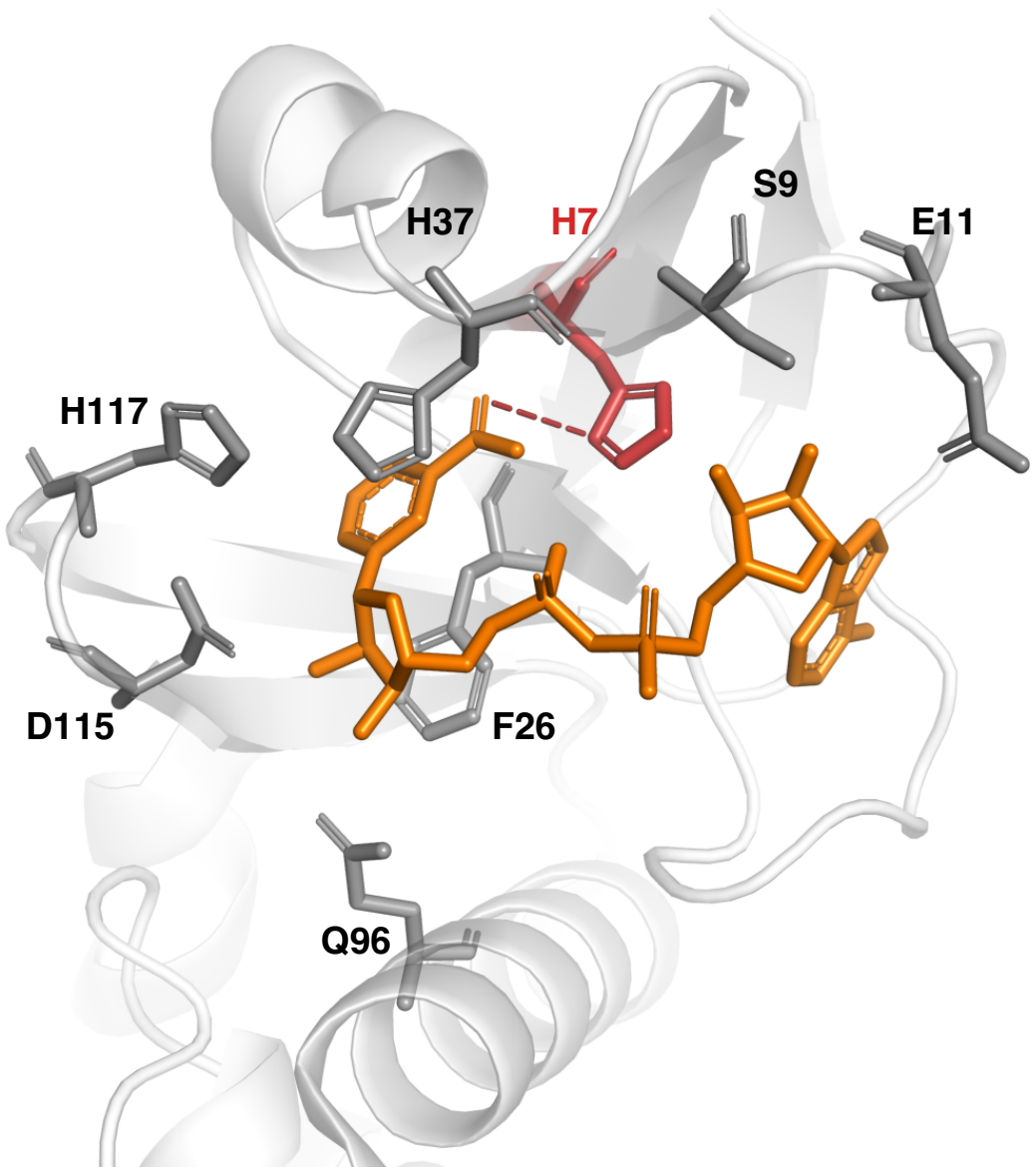

Figure S3: NAD-binding pocket of AcrIF11 (PDB 6KYF chain A). The NAD ligand (orange sticks) is shown together with the eight NAD-binding residues (Niu et al., 2020). H7, identified as an extreme outlier in §3.7 of the main paper (robust $z = +14.62$), is highlighted in red sticks; its hydrogen-bond contact with the ADP-ribose moiety of NAD is shown as a red dashed line. The remaining seven NAD-binding residues (S9, E11, F26, H37, Q96, D115, H117) are shown as grey sticks; none reached the outlier threshold $z \geq 3$ in this analysis (full ranking in Table S6).

S5 Per-family analysis details

This section supports §3.5 of the main paper. All quantities pool the five outer folds, so every one of the 232 P40 positives contributes exactly one held-out score.

S5.1 Per-family Recall@K

Table 6 of the main paper compares AcrSeek with the AcrNET(40%) reference on AUROC, the one ranking measure the baseline's saturated scores admit. Table S9 gives the corresponding Recall@K for the three AcrSeek encoders — the share of a family's positives falling in the top K of their own fold, K being that fold's positive count. The two views answer different questions:

AUROC asks how far up the ranking a family's members sit, Recall@K whether they reach the operating point at which the main results are reported, and the second is the more severe of the two.

Table S9: Per-family Recall@K, pooled over the five outer folds, for families with more than one P40 member.

Family	n	ESM-1b	ESM-2 650M	ESM-2 3B
AcrIIA7	53	0.698	0.925	0.943
AcrID1	21	0.381	0.667	0.476
AcrIIA8	19	0.947	1.000	0.947
AcrIIA9	18	0.556	0.778	0.778
AcrIF11	14	0.286	0.714	0.857
AcrIF6	9	0.111	0.000	0.222
AcrIIC1	7	0.000	0.000	0.000
AcrIIA11	4	0.000	0.000	0.250
AcrIF9	4	0.000	0.250	0.000
AcrVIB	3	0.333	0.333	0.000
AcrIIA19	2	1.000	1.000	0.500
AcrIF8	2	0.000	0.000	0.000
AcrIIA2	2	1.000	1.000	0.000
AcrIIC2	2	0.000	0.000	0.000

The two measures can diverge. AcrIF6 reaches AUROC 0.829–0.915, above most negatives, yet its Recall@K is at most 0.222: it is ranked respectably but rarely reaches the top of the list, whereas AcrIIC1 is low on both and is not separated from the negatives at all.

S5.2 Type and subtype groupings

Table S10 reports the coarser groupings by targeted CRISPR type and subtype. These aggregate families of very unequal size — subtype II-A alone accounts for 114 of the 232 positives, 53 of them from AcrIIA7 — so a group figure largely reflects its dominant family, and we treat the per-family decomposition of Table 6 in the main paper as the primary result. The contrasts are nonetheless clear: detection of type II Acrs exceeds type I under every encoder, and within type II, subtype II-A is recovered while II-C is not.

S5.3 Seen and unseen families

Of the 86 Acr families, 72 contribute a single sequence to P40, so holding that sequence out leaves no member of its family in training; we call these unseen-family positives. They are unfamiliar by more than family label: 70 of the 72 have no significant BLAST homology ($E \geq 10^{-3}$) to any training positive. Table S11 separates them from the 160 whose families are represented in training. *Micro* pools all positives and *macro* averages the per-family rates first, so the gap between them measures how far a pooled figure is carried by the largest families.

The AUROC in Table S11 establishes that unseen-family positives outrank most negatives, but says nothing about whether a screen could act on that. Table S12 therefore reports their recall against the number of top-ranked candidates, with chance as the reference.

AcrNET(40%) is not tabulated there. Its positive-class probability saturates over blocks of up to 1,640 sequences that it cannot order internally, and for candidate lists of 500 or fewer none of its unseen-family positives falls unambiguously inside or outside the cut: every one of the 23 that could be recovered at those depths sits in a block the list boundary crosses. A figure there would therefore be an expectation over an arbitrary tie-break rather than a recall the

Table S10: Recall@K by targeted CRISPR type and subtype, pooled over the five outer folds. Groups with fewer than three positives are omitted.

Group	n	ESM-1b	ESM-2 650M	ESM-2 3B
Type				
Type II	126	0.563	0.690	0.683
Type I	89	0.146	0.292	0.348
Type VI	10	0.100	0.100	0.000
Type V	5	0.000	0.000	0.000
Subtype				
II-A	114	0.623	0.763	0.754
I-F	49	0.102	0.224	0.347
I-D	21	0.381	0.667	0.476
II-C	12	0.000	0.000	0.000
I-C	9	0.000	0.111	0.222
I-E	9	0.000	0.000	0.222
VI-A	7	0.000	0.000	0.000
V-A	5	0.000	0.000	0.000
VI-B	3	0.333	0.333	0.000

Table S11: Detection by regime, AcrSeek (ESM-2 650M). Recall@K with Wilson 95% intervals.

Regime	n	Families	Micro	95% CI	Macro	AUROC
Seen-family	160	14	0.700	[0.62, 0.77]	0.476	0.954
Unseen-family	72	72	0.028	[0.01, 0.10]	0.028	0.797

method attains, and would not be comparable with AcrSeek, whose largest tie block among these positives is 2. This is the same limitation that leads Table 6 of the main paper to compare the methods on AUROC, which is unaffected because it resolves a tie by crediting half of it to each ordering, so a saturated block contributes its expected value by construction rather than by an arbitrary choice.

Table S12: Unseen-family recall (72 positives) against the number of top-ranked candidates per fold, out of 17,808 test sequences.

Method	47	100	250	500	1,000	2,000	5,000
Chance	0.003	0.006	0.014	0.028	0.056	0.112	0.281
AcrSeek (ESM-1b)	0.028	0.125	0.153	0.292	0.417	0.542	0.708
AcrSeek (ESM-2 650M)	0.028	0.083	0.181	0.264	0.375	0.500	0.694
AcrSeek (ESM-2 3B)	**0.125**	**0.181**	**0.278**	**0.319**	**0.431**	**0.542**	**0.708**

S5.4 Family geometry in the projection space

If families formed separate compact clusters, a single positive centroid would be the wrong summary and additional prototypes the natural repair. Table S13 tests that premise on the trained projection heads without retraining. *Spread* is the mean pairwise distance between a family's members and *displacement* the distance from its centroid to the global positive centroid; both centroids are unit-normalised, since the mean of unit vectors shrinks towards the origin in proportion to the spread and would otherwise understate the displacement of exactly the

dispersed families at issue. A ratio above one would indicate a compact group sitting apart from the centroid.

Table S13: Family geometry, 5-fold means, for families with at least five P40 members. Displacement/spread below one indicates a family more dispersed within itself than displaced from the global centroid.

	**Centroid distance**			**Spread**			**Displ./spread**		
**Family**	**1b**	**650M**	**3B**	**1b**	**650M**	**3B**	**1b**	**650M**	**3B**
AcrIIA7	0.852	0.495	0.530	0.672	0.493	0.424	0.279	0.280	0.425
AcrID1	1.140	1.009	1.160	0.829	0.929	0.705	0.369	0.362	0.457
AcrIIA8	0.570	0.575	0.476	0.490	0.526	0.373	0.478	0.372	0.595
AcrIIA9	1.041	0.812	0.662	0.787	0.792	0.495	0.364	0.368	0.472
AcrIF11	1.162	0.812	0.642	0.831	0.752	0.485	0.403	0.402	0.511
AcrIF6	1.371	1.255	1.375	0.971	1.150	0.856	0.481	0.544	0.473
AcrIIC1	1.597	1.470	1.683	1.034	1.223	0.936	0.451	0.641	0.471

The families that resist detection are the distant ones — AcrIIC1 and AcrIF6 hold the two largest centroid distances under every encoder — but none of them is compact. The displacement-to-spread ratio stays below one for every family and encoder (0.28–0.64 as family means, and below one in each individual fold), so the undetected families are scattered as well as distant, and a second prototype placed at a family mean would not obviously reach them.

S6 Classification-head comparison

The classification-head setting of Table 5 of the main paper keeps the projection head of §2.2 unchanged and changes how its output is scored: the unit-norm projection $\mathbf{z} \in \mathbb{R}^{256}$ is passed through a linear layer to a single logit, which a sigmoid maps to a probability; the head is trained by cross-entropy, in place of the combined triplet–focal objective of §2.3. Training uses the whole S_i-Train-Sub set, shuffled each epoch into batches of 2,048, so the imbalance is present in every batch and is left for the objective to handle.

The learning rate (3×10^{-5}) and projection dimension (256) were taken unchanged from the ESM-2 650M configuration of the main model. Holding them fixed is what confines the comparison in Table 5 of the main paper to the scoring rule and the objective; its cost is that a learning rate suited to a cross-entropy objective, rather than to the metric-learning one, was not sought. The search reported at the end of this section addresses that cost directly. What was searched here is the parameter that governs the imbalance correction itself, since leaving it untuned would not have tested whether a conventional classifier can be adapted to the imbalance. We searched the positive-class weight over six values spaced logarithmically from 1 to 383, $\{1, 3, 10, 30, 100, 383\}$, the upper end matching the negative-to-positive ratio, so that the range within which a re-weighting scheme could plausibly help is covered at roughly threefold intervals rather than sampled only at its ends. Selection followed the Shared HP procedure of §S1 without modification: the combination with the highest mean smoothed validation AUPRC across the five inner folds, with the epoch and the decision threshold set to the medians of the per-fold optima. Validation was recorded at 100 candidate epochs, as for the main model.

The bias of the linear layer was initialised to the logit of the training positive rate, following Lin et al. (2017). Left at its default the initial loss on a 1:383 batch is dominated by negatives and the run spends its early epochs recovering from that, which would understate the baseline for a reason unrelated to its merits.

Table S14 reports the search. Up-weighting the positive class did not help: the selected weight is 1, that is, the unweighted loss, and validation AUPRC falls monotonically as the weight rises

across all six values, from 0.4286 at a weight of 1 to 0.3639 at 383. The selection is therefore not an artefact of sampling only the ends of the range. Training ran for up to $E_{\max} = 2{,}000$ epochs and the selected epoch was 280, so neither the searched range nor $E_{\max}$ limited the outcome.

Table S14: Positive-class weight search for the classification head. Values are the mean across the five inner folds of each setting's best smoothed validation AUPRC. The selected row is in bold; the epoch and threshold are medians of the per-fold optima.

Positive-class weight	Val AUPRC	Epoch	Threshold
1	**0.4286**	**280**	**0.125**
3	0.4280	320	0.192
10	0.4187	1,020	0.986
30	0.3988	1,160	0.981
100	0.3819	940	0.926
383	0.3639	620	0.538

The learning rate best suited to a cross-entropy objective need not be the one selected for the metric-learning model, so we repeated the search with the learning rate as a second axis. The grid spanned six rates from 3×10^{-6} to 1×10^{-3} at roughly threefold intervals, crossed with the same six positive-class weights, giving 36 combinations evaluated on the same five inner folds under the same selection rule and the same $E_{\max}$. Table S15 reports the mean smoothed validation AUPRC of every combination.

Table S15: Joint search over the learning rate and the positive-class weight for the classification head. Values are the mean across the five inner folds of each combination's best smoothed validation AUPRC. The selected combination is in bold.

	Positive-class weight					
Learning rate	1	3	10	30	100	383
3×10^{-6}	0.4295	0.4227	0.4100	0.3729	0.3037	0.1854
1×10^{-5}	**0.4312**	0.4250	0.4210	0.4040	0.3663	0.3447
3×10^{-5}	0.4286	0.4280	0.4187	0.3988	0.3819	0.3639
1×10^{-4}	0.4257	0.4082	0.3852	0.3622	0.3539	0.3328
3×10^{-4}	0.3886	0.4113	0.4034	0.3827	0.3837	0.3784
1×10^{-3}	0.4260	0.4177	0.4149	0.4000	0.4090	0.4004

Three features of this surface bear on the comparison. The unweighted loss remains preferred at five of the six rates; only at 3×10^{-4} does a weight of 3 edge ahead, and that rate is the weakest of the six at its own best weight. The response to the learning rate is shallow: setting that rate aside, the best value attained at each of the remaining five lies between 0.4257 and 0.4312, a spread of 0.0055, against a fold-to-fold standard deviation of 0.0356 for the selected setting itself. The selected combination is interior to the grid on both axes.

Applying the same selection rule to this grid returns a learning rate of 1×10^{-5} with an unweighted loss, epoch 680 and threshold 0.025, at a mean validation AUPRC of 0.4312 against 0.4286 for the inherited rate. Re-running the outer loop under that configuration gives a test AUPRC of 0.4305 (standard deviation 0.0341 across folds), against 0.4301 (0.0384) for the configuration of Table 5 of the main paper. The paired difference is +0.0004, with a fold-to-fold standard deviation of 0.0146 and a 95% confidence interval of $[-0.018, +0.019]$. The margin of the combined loss over the classification head is accordingly 0.0369 with the searched rate and 0.0373 with the inherited one; under either configuration the combined loss leads in all five folds. Tuning the learning rate independently therefore does not alter the comparison, and Table 5

retains the configuration that holds the optimisation settings fixed, so that its rows differ only in the scoring rule and the objective.

Because the selected rate lies at neither end of the grid, the remaining question is whether the lowest rate was limited by $E_{\max}$ rather than by performance. At 3×10^{-6} the selected epoch reached $E_{\max}$ in one of the five folds. Re-running the two lowest rates with $E_{\max}$ raised threefold, to 6,000, leaves every fold's optimum interior to the allowance, and 3×10^{-6} still does not overtake 1×10^{-5} (0.4308 against 0.4312). The lower edge of the grid is therefore closed on performance rather than on the epoch allowance.

References

Y. Li, Y. Wei, S. Xu, Q. Tan, L. Zong, J. Wang, Y. Wang, J. Chen, L. Hong, and Y. Li. Acrnet: predicting anti-crispr with deep learning. *Bioinformatics*, 39(5):btad259, 2023.

T.-Y. Lin, P. Goyal, R. Girshick, K. He, and P. Dollar. Focal loss for dense object detection. In *Proceedings of the IEEE International Conference on Computer Vision (ICCV)*, Oct 2017.

Y. Niu, L. Yang, T. Gao, C. Dong, B. Zhang, P. Yin, A.-K. Hopp, D. Li, R. Gan, H. Wang, X. Liu, X. Cao, Y. Xie, X. Meng, H. Deng, X. Zhang, J. Ren, M. O. Hottiger, Z. Chen, Y. Zhang, X. Liu, and Y. Feng. A type i-f anti-crispr protein inhibits the crispr-cas surveillance complex by adp-ribosylation. *Mol Cell*, 80(3):512–524.e5, 2020.
